# Telomerase RNAs in land plants

**DOI:** 10.1093/nar/gkz695

**Published:** 2019-08-08

**Authors:** Petr Fajkus, Vratislav Peška, Michal Závodník, Miloslava Fojtová, Jana Fulnečková, Šimon Dobias, Agata Kilar, Martina Dvořáčková, Dagmar Zachová, Ivona Nečasová, Jason Sims, Eva Sýkorová, Jiří Fajkus

**Affiliations:** 1 Department of Cell Biology and Radiobiology, Institute of Biophysics of the Czech Academy of Sciences, v.v.i., Brno CZ-61265, Czech Republic; 2 Laboratory of Functional Genomics and Proteomics, NCBR, Faculty of Science, Masaryk University, Brno CZ-61137, Czech Republic; 3 Mendel Centre for Plant Genomics and Proteomics, CEITEC, Masaryk University, Brno CZ-62500, Czech Republic; 4 Max Perutz Labs, University of Vienna, Dr. Bohr Gasse 9, A-1030, Vienna, Austria

## Abstract

To elucidate the molecular nature of evolutionary changes of telomeres in the plant order Asparagales, we aimed to characterize telomerase RNA subunits (TRs) in these plants. The unusually long telomere repeat unit in *Allium* plants (12 nt) allowed us to identify TRs in transcriptomic data of representative species of the *Allium* genus. Orthologous TRs were then identified in Asparagales plants harbouring telomere DNA composed of TTAGGG (human type) or TTTAGGG (*Arabidopsis*-type) repeats. Further, we identified TRs across the land plant phylogeny, including common model plants, crop plants, and plants with unusual telomeres. Several lines of functional testing demonstrate the templating telomerase function of the identified TRs and disprove a functionality of the only previously reported plant telomerase RNA in *Arabidopsis thaliana*. Importantly, our results change the existing paradigm in plant telomere biology which has been based on the existence of a relatively conserved telomerase reverse transcriptase subunit (TERT) associating with highly divergent TRs even between closely related plant taxa. The finding of a monophyletic origin of genuine TRs across land plants opens the possibility to identify TRs directly in transcriptomic or genomic data and/or predict telomere sequences synthesized according to the respective TR template region.

## INTRODUCTION

The origin of linear chromosomes associated with divergence of eukaryotes led to the evolution of mechanisms counteracting the incomplete replication of chromosome ends—the telomeres. The most common mechanism to overcome the end-replication problem involves a ribonucleoprotein complex enzyme—telomerase. Telomerase elongates the 3′-end of telomeric DNA using the catalytic activity of its core protein subunit—telomerase reverse transcriptase (TERT) - which can repeatedly add a short DNA stretch to telomeric DNA (1). The sequence added by telomerase is directed by a template region in telomerase RNA (TR), the other core telomerase subunit (2). In addition to these two core subunits, the complex of telomerase involves several other associated proteins which affect various steps of telomerase function *in vivo*, as, e.g. telomerase assembly, trafficking, localisation, processivity, or its recruitment to telomeres (3,4), (see also (5–7) for recent reviews).

Due to the mechanism of their elongation by telomerase, telomere DNAs are formed by tandemly repeated arrays of short DNA sequence units, which are usually well conserved through large taxa of higher eukaryotes, as, e.g. (TTAGGG)_*n*_ in vertebrates (8). Lower eukaryotes show more extensive diversity in telomere DNA, as can be exemplified in yeast or algal telomeres (9–12). Finally, organisms which had acquired some of the telomerase-independent mechanisms of telomere synthesis, either during evolution or due to targetted disruption of telomerase, can possess entirely different telomeres composed of retrotransposons or tandem repeats (for review, see (13)).

Among land plants, telomeres of (TTTAGGG)*_n_* sequence were first characterized in Arabidopsis (14) and then identified in many other land plant species. However, numerous exceptions were found as well, first in *Allium* and related Allioidae plant species (15,16), then among the other Asparagales genera (17–20) and other plant taxa (21–23). In our previous studies, we demonstrated that changes in telomere sequences correspond to the phylogenetic divergence of plant families and genera. Thus, in monocotyledonous plants, telomere DNAs evolved from (TTTAGGG)_*n*_ (common also in plants of the Asparagales order up to the family *Doryanthaceae*) and a switch to (TTAGGG)_*n*_ occurred with the divergence of the family *Iridaceae* (20,24). Finally, (CTCGGTTATGGG)*_n_* telomeres emerged with the divergence of the *Allium* genus (25). With characterisation of *Allium* telomeres, we elucidated the last known exception from canonical telomeres among land plants and demonstrated that all these ‘unusual’ plant telomeres are synthesized by telomerases. The story of unusual plant telomeres could, therefore, finish almost symbolically at the *Allium* genus, where it began in 1995 (15). However, this would leave some fundamental questions open. The first of these is the molecular basis of the evolutionary switches in telomere DNA sequences. In this work, we approach this question and consider the following possible scenarios: (i) TR remained essentially the same across the Asparagales phylogeny, and the observed switches in telomere synthesis occurred either as a result of mutations in the template region of TR or mutation in the vicinity of the template sequence which could change the boundaries of the region recognized as a template; (ii) different RNA molecules took over the TR function. Generally, characterization of TRs is complicated by their extreme divergence in size (from 159 nt in *Tetrahymena* (2) to 2200 nt in *Plasmodium* (26)), nucleotide sequence, and pathways of biogenesis among organisms. A certain level of conservation can be seen only at the level of the secondary structure motifs in TRs (reviewed in (27)). This complicates an *in silico* TR identification, and the only TR domain of at least partially predictable sequence is the template region which contains a permutation of the telomere repeat unit in a given organism elongated by at least one nucleotide.

To characterise TRs and their changes underlying evolutionary transitions of telomeres in Asparagales, we take advantage of the unusually large length of the *Allium* telomere repeat unit (12 nt) to identify candidate TRs in transcriptomes depleted of ribosomal RNA (rRNA) in multiple *Allium* species in parallel, and examine the candidate TRs by reconstitution experiments. Importantly, based on the *Allium* TRs, we identify their orthologs in the other land plants and find their template regions corresponding to their telomere repeat sequences. Interestingly, we also identify the corresponding homolog in *Arabidopsis thaliana*, where a different candidate TR, *TER1*, has been reported earlier to be able to provide a templating function in telomerase *in vitro* reconstitution experiments (28). However, we demonstrate here by *in vitro* and *in vivo* experiments that the newly identified TR is the natural templating subunit of telomerase in *Arabidopsis*, as well as are its orthologs in other flowering plants.

## MATERIALS AND METHODS

### Plant material

Seven-day-old seedlings were collected from *Allium nutans, A. ericetorum, A. angulosum* (Botanical Garden of the Faculty of Science of Masaryk University, Brno, Czech Republic), *A. cepa* cv. Všetana (SEMO a.s., Czech Republic), *A. fistulosum* cv. Bajkal (Nohel Garden a.s.). Root tips were collected from plants grown at the Institute of Biophysics, Brno, including *A. cepa* cv. Všetana, *Scilla peruviana* (29), *Cestrum elegans* (Brongn.) Schltdl. (21), *Tulbaghia violacea* (Rh plant s.r.o., Czech Republic), and from plants collected in fields, *A. ursinum* (Cernovický hájek nature reserve, Brno, Czech Republic). Seeds of *Nicotiana sylvestris* (TW 136, voucher: PI 555569) were a kind gift from Prof. Marie-Angèle Grandbastien (INRA, France). *Arabidopsis thaliana* T-DNA insertion line *tr*-1 (EKU19, FLAG_410H04; (30)) and wild type Wassilevskija (Ws-4) were obtained from the Versailles INRA collection (31). *A. thaliana* mutant line *tr*-2 (SALK_076745 and SALK_076745C) and wild-type accessions (ecotypes Col-0, N60000; Bela-1, N76696) were from the Nottingham Arabidopsis Stock Centre (32). Arabidopsis *tr* mutant lines and transformants generated for complementation studies were grown in growth chambers under conditions of 16 h light, 21°C and 8 h dark, 19°C, illumination 150 μmol m^−2^ s^−1^. Individual plants from each *tr* line were genotyped (see Supplementary Table S1 for primer sequences).

### RNA-seq library preparation

Total RNA was extracted from 100 mg of plant seedlings or root tips using the TRI-REAGENT (Sigma-Aldrich) according to the manufacturer's protocol. RNA samples from *A. nutans, A. cepa, A. ursinum, A. angulosum, A. ericetorum, A. fistulosum, Tulbaghia violacea, Scilla peruviana*, and *Cestrum elegans* were checked for quality (estimated from RIN–RNA integrity number ≥ 7.5) on an Agilent 2200 TapeStation system (Agilent Technologies) using RNA ScreenTape^®^ (Agilent Technologies) and RNA concentration was determined by a Qubit 2.0 fluorometer using a Qubit™ RNA BR Assay Kit. Ribosomal RNAs were depleted from 5 μg of total RNA of each sample by Ribo-Zero rRNA Removal Kit (Plant Leaf) and Ribo-Zero rRNA Removal Kit (Seed, Root) (Illumina^®^) used for seedlings and root tip samples, respectively. After rRNA depletion, samples were diluted in 10 μl of RNase-free water and 5 μl of each sample were used for RNA library construction using NEBNext^®^ Ultra™ II Directional RNA Library Prep Kit for Illumina® (NEB) according to the manufacturer's instructions, where the step for Poly(A) enrichment was omitted. Libraries were purified for a fragment size range from ∼200 to ∼500 bp by double-size selection using AGENCOURT^®^AMPURE^®^XP magnetic beads (Beckman Coulter) and library quality was checked by an Agilent 2200 TapeStation system using High Sensitivity D1000 ScreenTape^®^ (Agilent Technologies). Libraries were sequenced in CF Genomics (CEITEC MU) on a NextSeq500 platform (Illumina^®^) using NextSeq 500 v2.5. Mid Output 150 cycles kits (Illumina^®^) for 2 × 75 bp PE reads. To evaluate reliability of our RNA-seq datasets for telomerase RNA subunit identification, we investigated the occurrence of *TERT* transcripts in raw RNA-seq data (Supplementary Table S2). RNA-seq data generated in this project are available at NCBI (BioProject PRJNA542932).

### Transcriptome *de novo* assembly and filtering of *Allium* TR candidates

**Figure 1.**
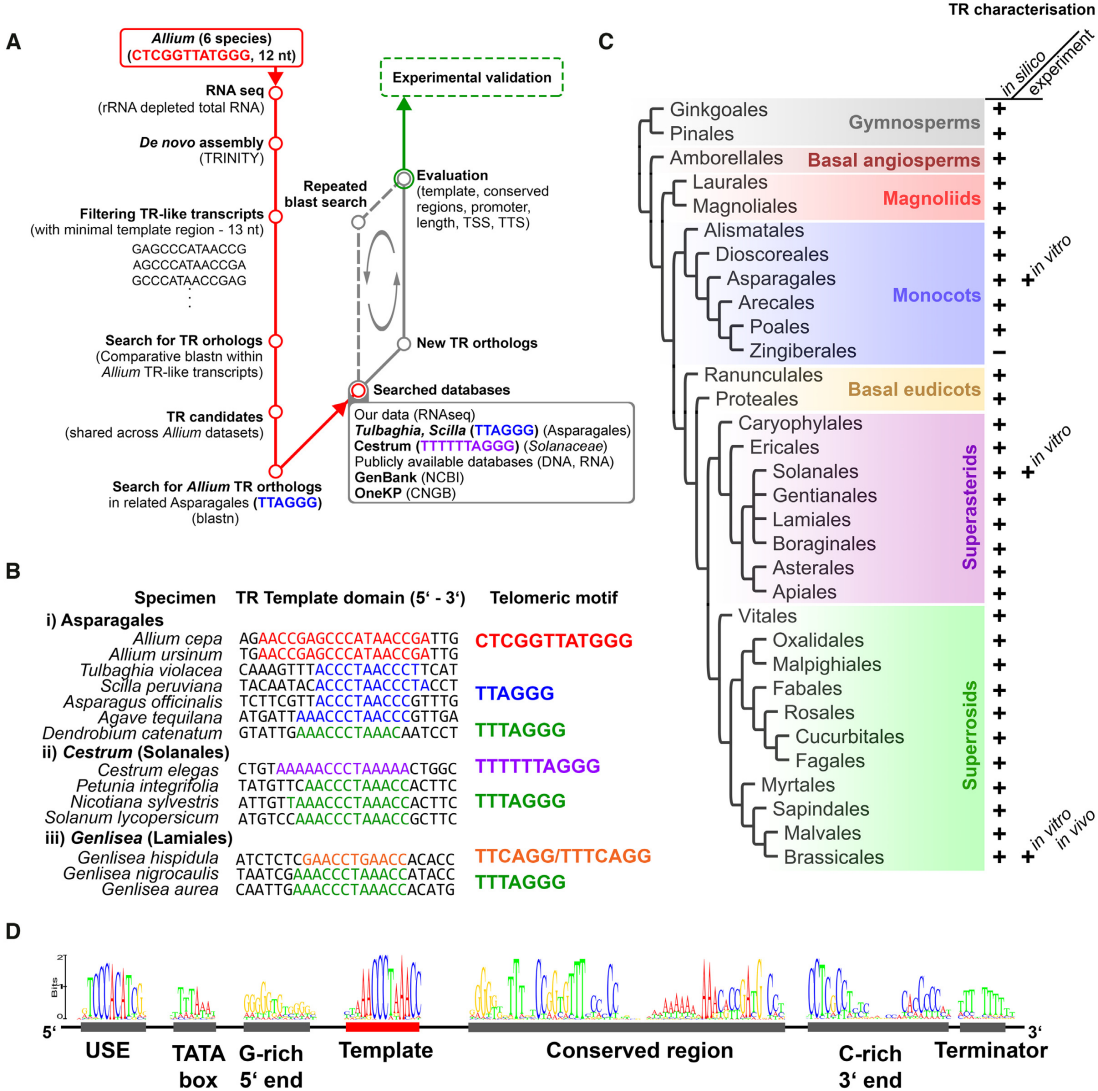
Identification of candidate TRs *in silico*. (**A**) Workflow for TR identification by comparative RNA sequencing and ortholog searching. (**B**) Putative template TR regions identified in plants with diverse telomere DNA motifs. Note the correspondence between the TR Template domain (left) and the telomeric motif (right). (**C**) An overview of identified TRs (+ sign) across major clades of land plants. Except for Zingiberales, putative TRs were identified in all representative plant orders (Supplementary Table S3). (**D**) Shared sequence motifs in plant TR genes. USE = Upstream Sequence Element. Sequence logo of each motif obtained from alignment of all identified TRs (Supplementary Figure S1).

RNA-seq *de novo* assembly was done using Trinity-v2.7.0 (https://github.com/trinityrnaseq/trinityrnaseq/wiki; (33)). The assembly was done with options for stranded RNA-seq with paired-end fastq data (Trinity –seqType fq –left inputfile_R1.fastq –right inputfile_R2.fastq –SS_lib_type RF –CPU 10 –max_memory 100G –bflyCPU 10 –KMER_SIZE 25 –output trinity_ouput). Putative TR transcripts possessing the minimal template region according to their telomere repeat motif were filtered out using grep, a LINUX command-line utility (https://www.gnu.org/software/grep/) (Figure 1A). For example, in the case of *T. violacea* transcripts containing one of the six possible template regions were filtered from the Trinity output (grep -E -A1 ‘(CCCTAAC|CCTAACC|CTAACCC|TAACCCT|AACCCTA|ACCCTAA)’ trinity_output.fasta > tulbaghia_TR_candidates.fasta). The standalone BLAST 2.2.28+ package (downloaded from NCBI; https://blast.ncbi.nlm.nih.gov/Blast.cgi) with the default parameters was used to compare datasets of putative TR transcripts all-to-all with the following BLASTN parameters (-word_size 7; -gapopen 5; -gapextend 2; -penalty −3; -reward 2) (34–36).

### Identification of TR orthologs outside *Allium*

Using *A. cepa* or *A. ursinum TR* transcript as a query in BLASTN, we searched for *Allium* TR homologs in our RNA-seq data from *T. violacea* (Amaryllidaceae), *S. peruviana* (Asparagaceae), and in selected transcriptomic and/or genomic data from Asparagales species (*Asparagus officinalis, Rhodophiala pratensis, Nolina bigelovii*, and *Agave tequilana*, accessions in Supplementary Table S3) available at NCBI (www.ncbi.nlm.nih.gov) or 1KP (www.onekp.com; (37–40)). BLAST runs with parameters as above, except for variations in Match/Mismatch scores (2/–3 and 1/–1), were used. Blast hits were evaluated according to sequence similarity and presence of conserved regions including the putative template domain region. Subsequently, new TR orthologs were identified in available datasets (accessions in Supplementary Table S3) gradually, going from closely related to evolutionarily distant plant species using novel TR candidates in BLAST searches (Figure 1A). The final set of TR homologs presented in this work (Supplementary Figure S1) was aligned in Geneious 8.1.9 (https://www.geneious.com) using MAFFT alignment (Algorithm: E-INS-I; Scoring matrix: 200PAM/*k* = 2; gap open penalty: 1.53) (41).

### Analysis of AcTR transcripts with northern-blot hybridisation

Total RNA was isolated from 7-day-old *A. cepa* seedlings using the TRI-REAGENT (Sigma-Aldrich) according to the manufacturer's protocol. For Northern blotting, samples of 10 μg and 20 μg of total RNA were separated on a 1.8% w/v formaldehyde–agarose gel. The gel was stained with Gel Star™ Nucleic Acid Gel Stain (10 000×, Lonza) and RNA was transferred onto a Hybond™ -XL membrane (GE Healthcare Amersham™) using sterile RNA transfer buffer (0.01 M NaOH, 3 M NaCl). The AcTR probe was radioactively labelled using the Decalabel DNA labeling kit protocol (Thermo Scientific). The membrane was hybridized overnight at 55°C with the [^32^P]-labelled AcTR probe and signals were visualized using a PhosphoImager FLA-7000 (Fujifilm).

### Pull-down assays

Preparation of protein extracts from 7-day-old *A. cepa* seedlings and TRAP assays were performed as described previously (42,43). Four μg of Dyskerin (H-3) antibody (Santa Cruz Biotechnology) diluted in HEPES buffer pH 7.5 (25 mM HEPES; 150 mM KCl; 5 mM MgCl_2_; 10% glycerol; 1% BSA; 0.1% Nonidet NP-40; 1 mM DTT; 0.1 mM PMSF; 0.6 mM ribonucleoside vanadyl complex; 1 μg.ml^−1^ pepstatin A; 2 μg.ml^−1^ leupeptin) was incubated with 600 μg of magnetic Dynabeads^®^Protein G (Invitrogen) overnight with rotation at 4°C. The Dyskerin(H-3)-conjugated Dynabeads^®^ were washed with HEPES buffer and twice with buffer W (used for preparation of protein extracts, (43)), and then resuspended in 400 μl of protein extract containing 1 mg of total protein. After 2 h incubation at 4°C, the unbound fraction was collected and the Dynabeads^®^ with immunoprecipitated protein complexes were washed 3 times with buffer W. In the last washing step, the bead suspension was divided into two equal aliquots, for RNA isolation and for TRAP assay (see below). The first aliquot of Dynabeads^®^ with immunoprecipitated protein complexes was washed with HEPES buffer and used for RNA isolation with TRI REAGENT (Molecular Research Center, Inc.). The RNA sample was reverse transcribed to cDNA by M-MuLV reverse transcriptase (New England Biolabs) in a 20 μl reaction primed by random nonamers (Sigma-Aldrich). The AcTR was detected by PCR with primers qAcTR_F and qAcTR_R (Supplementary Table S4), using Kapa Taq DNA Polymerase (Elisabeth Pharmacon) and 1 μl of cDNA as a template. PCR was performed as follows—95°C for 2 min, 28/30/32 cycles at 95°C for 20 s, 57°C for 30 s, 72°C for 30 s, and final extension 72°C for 5 min. As a mock control, a protein sample was processed in parallel in the same procedure without Dyskerin (H-3) antibody. The second aliquot was washed with TRAP reaction buffer, beads were resuspended in 47 μl of TRAP reaction buffer, and 1 μl of 10 μM TS21 substrate primer was added. TRAP assay was performed with VRP058 reverse primer (Supplementary Table S1).

### Quantitative analysis of *TR* transcripts

RNA was isolated using the TRI REAGENT (Molecular Research Center, Inc.) and purified by DNaseI treatment (TURBO DNA-free, Thermo Fisher Scientific). One μg of RNA was reverse transcribed to cDNA by M-MuLV reverse transcriptase (New England Biolabs) in a reaction primed by random nonamers (Sigma-Aldrich). Quantitative PCR was performed using FastStart SYBR Green Master (Roche) and a Rotorgene6000 cycler and software (Qiagen). One μl of 2× diluted cDNA was added to the 20 μl reaction mix. Reactions were carried out in technical triplicates and biological triplicates under conditions as follows: 10 min at 95°C; 35 cycles of 30 s at 95°C, 30 s at 56°C (ubiquitin10) or 30 s at 57°C (AtTR, AcTR, AcTERT, AcAct), 30 s at 72°C (at the end of this step, fluorescence was measured); and final extension (3 min at 72°C). Signals of AtTR transcripts were related to the ubiquitin10 reference and AcTR/AcTERT transcripts were related to the actin reference (Genbank accession GU570135.2). Trancription in the respective tissues was related to that in 7-day-old seedlings using the ΔΔCt method (44). Sequences of primers are given in Supplementary Table S1.

### Preparation of *TERT* and *TR* constructs for reconstitution

We amplified candidate TR subunits from the putative transcription start site (TSS) to the transcription termination site (TTS) using specific primers designed for assembled sequences from *A. cepa* (accession ace_TRINITY_DN59687_c0_g1_i2), *S. peruviana* (accession sci_TRINITY_DN93444_c0_g2_i1), *N. sylvestris* (Genbank NW_009498048.1, 77924..77682) and *A. thaliana* (Genbank AB646770, (30)). The *AtTER1* (28) and the 256 bp long control construct bearing the 5′ region of the *AtCDT1a* gene were amplified from *A. thaliana* genomic DNA (ecotype Columbia). PCR was perfomed with Kapa Taq DNA Polymerase (Elisabeth Pharmacon) and respective genomic DNA as a template as follows: 35 cycles at 95°C for 15 s, 60°C for 15 s, 72°C for 15 s. PCR products were introduced into the pCRIITOPO vector (Invitrogen) and sequenced. Constructs bearing a mutated template region of *A. thaliana* and *N. sylvestris* TRs were prepared by mutagenesis of the respective wild-type TR plasmids using a Quick Change Lightning Site-Directed Mutagenesis Kit (Agilent Technologies) according to the manufacturer's instructions (primers are listed in Supplementary Table S1). For reconstitution experiments, *TERT* genes were inserted into the *Nco*I site of the pEPEX vector using an InFusion HD Cloning kit (Clontech). Briefly, *TERT* sequences were amplified with Phusion HF DNA polymerase (Finnzymes) using respective specific primers extended with an adaptor sequence specific to pEPEX, and plasmid DNA or cDNA as a template. The coding DNA sequence (cds) from the start to the stop codon of *A. cepa TERT* (Genbank accession KT381713.1) was amplified from seedling cDNA using Phusion HF DNA Polymerase and specific primers described in (25). PCR products were cloned into pCRIITOPO vector and after sequencing, the *AcTERT* sequence was subcloned into pEPEX. *N. sylvestris TERT* was amplified from seedling cDNA using Phusion HF DNA Polymerase and two sets of primers covering the N- and C-parts of NsTERT that overlap within the exon 9 region (45) and then were combined with pEPEX vector in three-fragment InFusion reactions. For *A. thaliana* and *S. peruviana TERT*, we subcloned cds of *AtTERT* (46) and *SpTERT* (29) into *Bam*HI/*Eco*RI sites in pBluescript KS+. The *AtTERT* plasmid was cut with *Bam*HI and *Cla*I or the SpTERT plasmid with *Xba*I and *Xho*I, and mixed in an InFusion reaction with a PCR fragment of pEPEX prepared using specific primers extended with an adaptor sequences corresponding to the *AtTERT* construct region, or to the corresponding pBluescript region, respectively. All *TERT* constructs were verified by sequencing. All primers used for *TERT* and *TR* cloning are listed in Supplementary Table S1.

### Preparation of *AtTR* constructs and *Agrobacterium*-mediated transformation

Constructs bearing the *AtTR* sequence with a putative native promoter and/or terminator region (see Results) were prepared using *A. thaliana* genomic DNA as a template, KAPA Taq DNA Polymerase, and specific primers under the PCR conditions described above. PCR products were cloned into pDONR/Zeo (Invitrogen) using Gateway technology. Constructs bearing an artificial U6.26 promoter sequence and an *AtTR* sequence were sequentially subcloned in pBluescript KS+ using two- and three-fragment InFusion reactions. Finally, the pU6.26::AtTR constructs were amplified from plasmids and cloned into pDONR/Zeo. After sequencing, all constructs were subcloned from their entry clones into the destination vector pFAST-G01 (47). Primers used for cloning are listed in Supplementary Table S1. The *Agrobacterium tumefaciens* strain GV3101 was used for transformation of *A. thaliana* homozygous *tr*-1 plants by the flower-dip method. Seeds positive for T-DNA insertion were selected according to their GFP signal (47) under a stereomicroscope (Olympus SZX16) and propagated.

### CRISPR/Cas9-mediated *TER1* deletion

In order to specifically delete a 267bp portion of the *TER1* gene including the template region (see Supplementary Figure S2) a CRISPR /Cas9 vector was constructed as described before (48). Two gRNAs were designed that bind at coordinates 26490360 and 26490627 of chromosome 1 (Supplementary Figure S2). All gRNAs were designed to contain at least 50% GCs and to be next to a PAM sequence. The T1 plants transformed with the Cas9 construct were selected by YFP expression in the seeds and evaluated for Cas9 functionality. This was achieved by PCR screening over both target regions within the *TER1* locus (primer sequences in Supplementary Table S1). Samples with any additional shorter band suggesting loss of the *TER1* target were verified by PCR product cloning and sequencing. Only T1 plants that showed deletion of the *TER1* region were further propagated and genotyped for loss of the *TER1* region. It was necessary to go through at least two plant generations to select for a homozygous *TER1* deletion and to confirm loss of the *Cas9* transgene.

### *In vitro* transcription and reconstitution of telomerase activity

We used 200 ng of PCR product amplified from respective pCRIITOPO constructs with a T7 primer and a specific reverse primer as a template in *in vitro* transcription reactions using a HiScribe T7 Quick High Yield RNA synthesis kit (New England Biolabs). PCR products were purified with a PCR Purification kit (Qiagen). We performed reactions with all TR plasmids and a pCDT1a control in the sense orientation with respect to the T7 promoter site in pCRIITOPO, and additionally with plasmids bearing AtTR and AtTER1 sequences in the antisense orientation. RNA was purified using TURBO DNA-free (Thermo Fisher Scientific) followed by clean-up with TRI Reagent or a Total RNA Mini kit (Plant) (Geneaid). RNA concentration was determined spectrophotometrically. Reconstitution of telomerase activity was performed in a rabbit reticulocyte lysate (RRL) using a TnT® Quick Coupled Transcription/Translation System (Promega). Briefly, 100 ng of each TERT plasmid was mixed with various amounts of the respective RNA (500, 50, 5 ng; or as otherwise stated) and with RRL mix in a final volume of 10 μl. Samples with up to 1000 ng RNA were also tested in the case of AtTER1 constructs. Samples were incubated at 30°C for 50 min and then assayed by TRAP immediately or stored at −80°C. *In vitro* transcription and reconstitution experiments were repeated independently at least three times.

### Telomere Repeat Amplification Protocol (TRAP) and analysis of reaction products

Preparation of protein extracts from 7-day-old plant seedlings and TRAP assays were performed as described previously (20,42,43). Briefly, 1 μl of 10 μM TS21 or CAMV substrate primer (Supplementary Table S1) were mixed with 1 μl of telomerase extract containing 50 ng of total protein, and incubated at 26°C for 45 min in 25 μl of reaction buffer to allow extension of substrate primer by telomerase. After extension, telomerase was heat-inactivated and 1 μl of 10 μM reverse primer (listed in Supplementary Table S1), and two units of DyNAzyme DNA Polymerase (ThermoScientific) were added to start the PCR step of the TRAP assay (35 cycles of 95°C for 30 s, 65°C for 30 s, 72°C for 30 s), followed by a final extension of 72°C for 5 min. Products were analyzed by electrophoresis on a 12.5% polyacrylamide gel and visualized after staining with GelStar Nucleic Acid Gel Stain (LONZA) using the LAS-3000 system (Fujifilm). A reaction without protein extract was used as a negative control in the case of analyses of plant mutants. TRAP assays in *in vitro* reconstitution experiments were performed according to the same protocol, except that KAPA Taq DNA Polymerase and 1 μl of RRL samples were used instead of a telomerase extract. RRL samples with TERT only and the RRL mix without plasmid or RNA were used as negative controls. Reaction products of TRAP assays were cloned directly in pCRIITOPO and sequenced as described in (20). Alternatively, TRAP products were purified from TRAP samples using Agencourt AMPure XP Magnetic beads (Beckman Coulter) or electroeluted from polyacrylamide gel as described in (25), cloned, and sequenced.

### TRF analysis of *tr* mutants

Telomere length analysis was performed as described earlier (49) by Southern hybridisation of terminal restriction fragments (TRF) produced by digestion with *Tru*1I restriction endonuclease (ThermoScientific). TRF signals were detected using the telomeric oligonucleotide (5′-GGTTTAGGGTTTAGGGTTTAGGGTTTAG-3′) end-labelled with [γ-^32^P]ATP using polynucleotide kinase (New England Biolabs). Median telomere lengths were calculated as the weighted median of telomere lengths from TRF intensity profiles (50).

## RESULTS

### Identification of candidate TRs in *Allium* species by comparative analysis of RNA-seq data

In our search for putative telomerase RNA subunits, we generated RNA-seq data of total RNA depleted of rRNA from *A. angulosum, A. cepa, A. ericetorum, A. fistulosum, A. nutans*, and *A. ursinum* (GenBank acc.: BioProject PRJNA542932, assemblies are available at: https://www.ibp.cz/local/data/telomeraserna/. To set a comparative *in silico* approach (Figure 1A), we took advantage of the long telomere repeat motif in *Allium* (12 bp, CTCGGTTATGGG) and the assumption that a template region of any telomerase RNA subunit must be longer by at least one nucleotide than the telomere repeat unit to allow a correct annealing of telomere DNA to the TR prior to its elongation. Thus, circular permutations of minimal template regions that were used for *in silico* analysis were by definition 13 nt long. The candidate sequences were identified subsequently in a set of transcripts assembled by the Trinity tool (33). As the first step, we pulled out only those transcripts that contained any permutation of the minimal template region from each RNA-seq dataset (Supplementary Table S2) using the grep utility (see Materials and Methods). Then in a search for putative *Allium* homologs of candidate sequences, we cross-investigated all subsets of transcripts using BLASTN and each candidate transcript sequence as a query. Transcripts that revealed positive hit(s) with candidates from any other *Allium* species were further classified according to the presence of candidate homologs in all or in some of the respective datasets (Supplementary Table S4). The final set of putative TRs resulted from candidate transcripts that shared sequence similarities in all datasets and thus presumably originated from *Allium* TR homologs (Figure 1A). We identified one transcript from *A. angulosum* and *A. ursinum*, two transcripts from *A. cepa* and *A. ericetorum*, three transcripts in *A. fistulosum* and seven transcripts in *A. nutans* (details in Supplementary Table S2). Comparison of interspecific sequence similarity among all the *Allium* transcripts showed 84.7% pairwise identity compared to the consensus sequence (Supplementary Figure S3), suggesting that these Trinity-assembled transcripts represent orthologs of the same candidate *Allium* TR. The final *Allium* TR candidates shared the 18 nt long sequence AACCGAGCCCATAACCGA representing a putative template region, which corresponds to 1.5 telomere repeat units. Moreover, *Allium* TRs shared a G-rich region at the 5′end, and a C-rich region near the 3′-end of the transcript. To identify putative transcription start site (TSS) and transcription termination site (TTS) of the full-length *A. cepa* TR transcript, we mapped final TR transcripts back to the original RNA seq data. A BLAST search identified 193 reads and 130 reads of Trinity-assembled transcripts matching to the 5′and 3′ends, respectively, thus supporting the AcTR length of 240 nucleotides.

### Search for TRs in Asparagales outside of the *Allium* genus

Encouraged by the identification of putative TRs in *Allium* species, we applied a similar approach (Figure 1A) to identify putative TRs in our newly-generated RNA-seq data from plants with human-type telomere motifs (TTAGGG)_*n*_—*Tulbaghia violacea* and *Scilla peruviana* (order Asparagales). The number of Trinity-assembled transcripts was similar to that of the *A. ursinum* dataset; however, the number of candidates with circular permutations of the minimal template region was much higher (Supplementary Table S2). For example, a subset of 65997 candidate transcripts was pulled out from *T. violacea* RNAseq data using six permutations of the minimal template region (CCCTAAC, CCTAACC, CTAACCC, TAACCCT, AACCCTA, ACCCTAA), in contrast to 80 transcripts found in the *A. ursinum* dataset (compare data in Supplementary Table S2). This corresponds to the shorter expected minimal template region (7nt) defined by a 6 bp long telomere motif extended by one nucleotide. Moreover, sequences with short telomere-like motifs often occur within promoter regions of genes representing putative regulatory elements (51,52). Subsets of candidate TR transcripts from *S. peruviana* and *T. violacea* were further analysed using AcTR sequence as a query in a BLASTN search. In parallel, we searched for putative orthologs of AcTR in publicly available genomic and/or transcriptomic data from various species of the order Asparagales and we cross-mapped candidate hits back to our datasets (Figure 1A). Finally, we identified six TR candidates in plant families of Asparagales that share a human-type of telomeric repeat, i.e. Amaryllidaceae (*T. violacea, Rhodophiala pratensis*) and Asparagaceae (*S. peruviana, Agave tequilana, Nolina bigelovii* and *Asparagus officinalis*) (Supplementary Table S3). Interestingly, the arrangement of template domain regions of candidate TRs of *S. peruviana* and *A. officinalis* (Figure 1B) fits to our prediction of the template region (ACCCTAAC) for these species based on previous experimental results (29). Moreover, template regions of candidate TRs of *A. tequilana* and *N. bigelovii* (Figure 1B, and Supplementary Figure S1) suggest that these telomerases can synthesize mixed telomere motifs of the human and *Arabidopsis* type. This corresponds to the previously observed mixed repeats in telomerase products and telomeres of several Asparagales species (20,29). As a proof of concept, we identified also putative TR orthologs from *Dendrobium catenatum* and *Phalaenopsis equestris* (Orchidaceae) which represent members of plant families of Asparagales sharing an *Arabidopsis* type of telomeric repeat. Thus finally, comparison of putative TRs from three plant groups from Asparagales clearly showed that the sequence of the template region corresponds to the expected type of telomeric repeat, either of the human, *Arabidopsis*, or onion type, synthesized by the respective telomerases (Figure 1B).

### Identification of orthologous TRs across land plants

In addition to identification of putative TRs in representative Asparagales species, we searched for corresponding TR orthologues in available datasets from representative monocot, eudicot and gymnosperm species (see Figure 1C for examples and Supplementary Table S3 for comprehensive results). Surprisingly, we identified candidate TRs in datasets from representative species of major clades across land plant phylogeny. Comparison of 75 candidate TR sequences (Supplementary Figure S1, Supplementary Table S3) revealed more conserved regions within transcribed parts that are shared in plant TRs and may be important for TR secondary structure, i.e. 5′-G-rich region, template domain, a conserved region downstream from the template domain, C-rich region, and an AT-rich region near the 3′-end (Figure 1D). Comparison of TR candidates found within genomic datasets enabled further identification of conserved regions of plant TR genes with putative regulatory functions, i.e. TATA box, Upstream Sequence Element (USE) and terminator (Figure 1D and Supplementary Figure S1). Thus, our results suggest that the TR gene is highly conserved in contrary to the very divergent TR genes found in animal, yeast or protozoan models. Another surprising result came from the model species *A. thaliana* in which we found an orthologous TR sequence corresponding to the long non-coding RNA (lncRNA) gene *R8* previously described for its functions in hypoxic stress (30). However, this candidate TR contains all conserved plant TR regions mentioned above and it possesses the sequence CTAAACCCT within the putative template region, clearly defining an *Arabidopsis* type of telomeric repeat with an additional 2 nt that can serve as an anchor site for telomerase annealing to telomeres.

### Plants with unusual telomere DNA repeats harbour TR orthologs with the corresponding template region sequences

The results obtained in the Asparagales order, where telomere repeats had evolved first from (TTTAGGG)n to (TTAGGG)n (20,24), and, subsequently from (TTAGGG)*_n_* to (CTCGGTTATGGG)*_n_* (25) were instrumental for demonstration of the molecular nature of these transitions, changes in the templating region of the corresponding *TR* genes. Therefore, we aimed to examine whether the other known ‘exceptions’, i.e. plants bearing telomere repeats other than the most common (TTTAGGG)*_n_*, also harbour TRs with corresponding template regions. These include *Genlisea hispidula* with intermingled (TTCAGG)n and (TTTCAGG)n motifs (while the other *Genlisea* species, e.g. *G. nigrocaulis*, harbour common (TTTAGGG)n telomeres) (23), and *Cestrum elegans* with (TTTTTTAGGG)n repeats synthesized by telomerase (53). Indeed, we were able to identify TRs harbouring the corresponding template regions in available datasets of *Genlisea* species (Figure 1B, Supplementary Table S3). Moreover, we generated and processed RNA-seq data of *C. elegans* (Supplementary Table S2), and we identified a candidate TR sharing conserved plant TR motifs. Comparison of *Genlisea*, Solanaceae and Asparagales TR subunits confirms that change of the telomere motif synthesized by their telomerases is dictated by sequence variation within and around the central motif ACCCTAA (Figure 1B).

### Experimental characterization of AcTR

**Figure 2.**
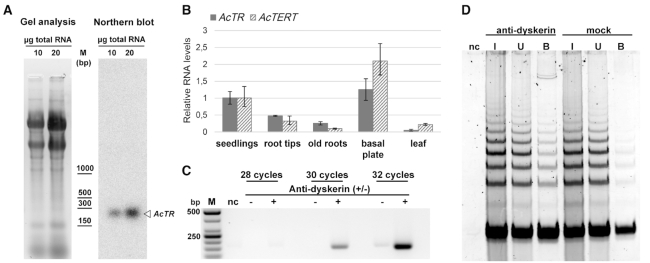
Experimental characterisation of AcTR. (**A**) Detection of AcTR using Northern blot hybridisation using 10 and 20 μg of total RNA, respectively. (**B**) Analysis of the levels of *AcTR* and *AcTERT* transcripts using RT-qPCR. Levels of AcTR and AcTERT mRNA in seedlings were arbitrarily set to 1 while the levels of AcTR in seedlings were 64 times higher than those of AcTERT mRNA based on their relative levels with respect to the actin reference. (**C**) RT-PCR detection of AcTR in *A. cepa* cell extracts from seedlings in a fraction immunopurified with anti-dyskerin antibody immobilized to Dynabeads (+) or Dynabeads without the antibody (–). (**D**) Telomerase activity was immunoprecipitated with the anti-Dyskerin antibody. The antibody was immobilized to G-protein Dynabeads and incubated with a protein extract from *A. cepa* seedlings (I, input). The unbound fraction was checked for the loss of telomerase activity during incubation (U, unbound). After washing, Dynabeads® with bound telomerase complex (B, bound) were directly used in TRAP assays. In parallel, the same procedure was done without antibody (mock) showing only a background activity. As a negative control (nc) buffer W was used in a TRAP assay.

As the first step of the experimental demonstration of AcTR functionality, we examined the occurrence of AcTR transcripts and possible alternatively spliced variants in *A. cepa*. Northern hybridization with a radioactively labelled AcTR probe showed a single RNA signal in onion seedlings (Figure 2A). Its length corresponds to the predicted size of the AcTR transcript deduced from RNA-seq data (see above). Moreover, this result suggests that the AcTR is present as a full length transcript only, i.e. without splicing or any other kind of extensive posttranscriptional truncation. In subsequent RT-qPCR analyses we found AcTR transcripts peaking in the basal plate and seedlings, the tissues with the maximum level of transcripts of the other core telomerase gene, *AcTERT* (Figure 2B).

The RNA subunit of mammalian telomerases associates with the protein Dyskerin (DKC1) which is important for the correct assembly of the active telomerase complex (reviewed in (54)). Our recent data suggest that this function is conserved also in plants (55). Therefore we examined whether Dyskerin associates with onion telomerase *in vivo*, and whether the identified AcTR is a component of a functional telomerase complex in *A. cepa*. We performed a pull-down experiment using *A. cepa* protein extract and Dyskerin (H-3) antibody. We observed that AcTR was clearly detected in the immunoprecipitated fraction by RT-PCR (Figure 2C). Moreover, telomerase activity was immunoprecipitated with the Dyskerin antibody immobilized to Dynabeads®Protein G as demonstrated by TRAP assay, while only a background activity associated with Dynabeads non-specifically (Figure 2D). These results indicate a wide conservation of a Dyskerin-TR association among plant and animal kingdoms, and the functionality of AcTR as a telomerase RNA component in *A. cepa*.

### Telomerase templating function of putative TRs from Asparagales

**Figure 3.**
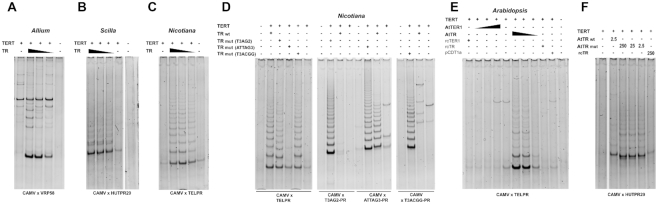
*In vitro* reconstitutions of telomerase activity using wt (A-C) and mutant or alternative TRs (D-F). (**A**) Serial dilutions of *A. cepa* wt TR; (**B**) Serial dilutions of *S. peruviana* wt TR; (**C**) Serial dilutions of *N. sylvestris* wt TR; (**D**) *N. sylvestris* wt and mutant TRs; (**E**) *A. thaliana* reconstitutions using TER1, AtTR, reverse complementary TER (rcTER), reverse complementary AtTR (rcTR), and CDT1a RNAs. Serial dilutions of AtTR and TER1 are shown. (**F**) comparison of reconstitutions using AtTR and serial dilutions of AtTR mut where the template region was mutated to produce human-type telomere repeats. rcTR was used as a control. Primers used in TRAP assays are shown below each panel.

Telomerase templating function was examined using *in vitro* reconstitution experiments with the identified TRs from *A. cepa* and *S peruviana*, and their corresponding TERT subunits characterized previously (25,29). In both cases, telomerase activity was reconstituted successfully in a rabbit reticulocyte lysate (RRL) (Figure 3A, B) producing ladders of the expected 12 and 6 nt periodicity, respectively, and with weak additional bands corresponding to the alternative modes of initial elongation of the substrate primer, as we described previously (25,29). Importantly, lack of the ladder pattern was observed in negative controls, i.e. in the absence of either the TR subunit or of both TR and TERT subunits in the reaction mixture. To confirm the telomerase-dependent origin of reaction products, the resulting TRAP products of *A. cepa* were cloned and sequenced. The results verified elongation of the substrate primer with typical 12-nt long repeats produced by reconstituted *Allium* telomerase (Supplementary Figure S4).

### Functional testing of TRs identified in model plants

As a proof of concept, we tested the putative templating function of predicted TR sequences from two plant models that were used in pioneering experiments for plant telomerase, *Nicotiana* and *Arabidopsis* (42,56). For *N. sylvestris* telomerase (Figure 3C), the functionality of NsTR was examined in reconstitution experiments *in vitro* and telomerase activity producing regular ladders of products in TRAP assays was demonstrated, while control reactions lacking either NsTR or both core telomerase subunits, NsTR and NsTERT, were clearly negative. Subsequent cloning and sequencing of the TRAP products confirmed elongation of the substrate oligonucleotide with variant numbers of TTTAGGG units (Supplementary Figure S4). In parallel, reconstitutions were performed with three different mutant versions of NsTR, in which the wild-type (WT) template region (5′-TAAACCCTAAACC-3′) has been changed to 5′-TAAACCTAAACC -3′, 5′-TAAtCCCTAAtCC-3′, or 5′-TAAACCgTAAACC-3′ (Figure 3D). All mutant NsTRs then produced corresponding types of telomere repeats—(TTTAGG)*_n_*, (ATTAGGG)*_n_* and (TTTACGG), respectively, as demonstrated by cloning and sequencing the resulting TRAP products produced with various combinations of substrate and reverse primers (Supplementary Figure S4).

Interestingly, the *A. thaliana* TR ortholog is different from the previously reported putative telomerase RNA subunits in *A. thaliana*, TER1 and TER2, of which TER1 was thought to provide telomerase templating function based on overexpression and reconstitution experiments (28). Therefore, both candidate telomerase RNA subunits—AtTR and TER1—were examined in parallel, first in *in vitro* reconstitution experiments with AtTERT. While AtTR was able to reconstitute telomerase activity in three successive 10x dilutions, TER1 did not provide any specific products at any of the corresponding concentrations (Figure 3E). As negative controls, we used RNA of AtTR and TER1 constructs transcribed in the antisense orientation and a construct bearing the 5′ region of the *CDT1a* gene that contains a telomere-like sequence CTAAACCCT, similar to the predicted TER1 template region (57) as well as to the AtTR template region (Supplementary Figure S1). Products of the TRAP assays of AtTR+AtTERT reconstitutions were cloned and sequenced, confirming the synthesis of a regular tandem repeat of TTTAGGG units (Supplementary Figure S4). In addition, a mutant version of AtTR bearing a point deletion of A within the template region to synthesize the human-type telomere repeat was also able to reconstitute telomerase activity producing corresponding bands with 6 nt periodicity in TRAP assays (Figure 3F) and the expected telomeric repeats in TRAP products (Supplementary Figure S4). To exclude the possibility that our *in vitro* reconstitution with TER1 failed because of technical problems, we prepared plant mutants with CRISPR/Cas9-induced deletion of *TER1* gene. Importantly, *TER1* gene deletion did not show any effect on telomerase activity in plants homozygous for *TER1* deletion (for details see Supplementary Figure S2).

### *The AtTR* gene is expressed in meristem tissues and *attr* mutants show progressive telomere shortening

**Figure 4.**
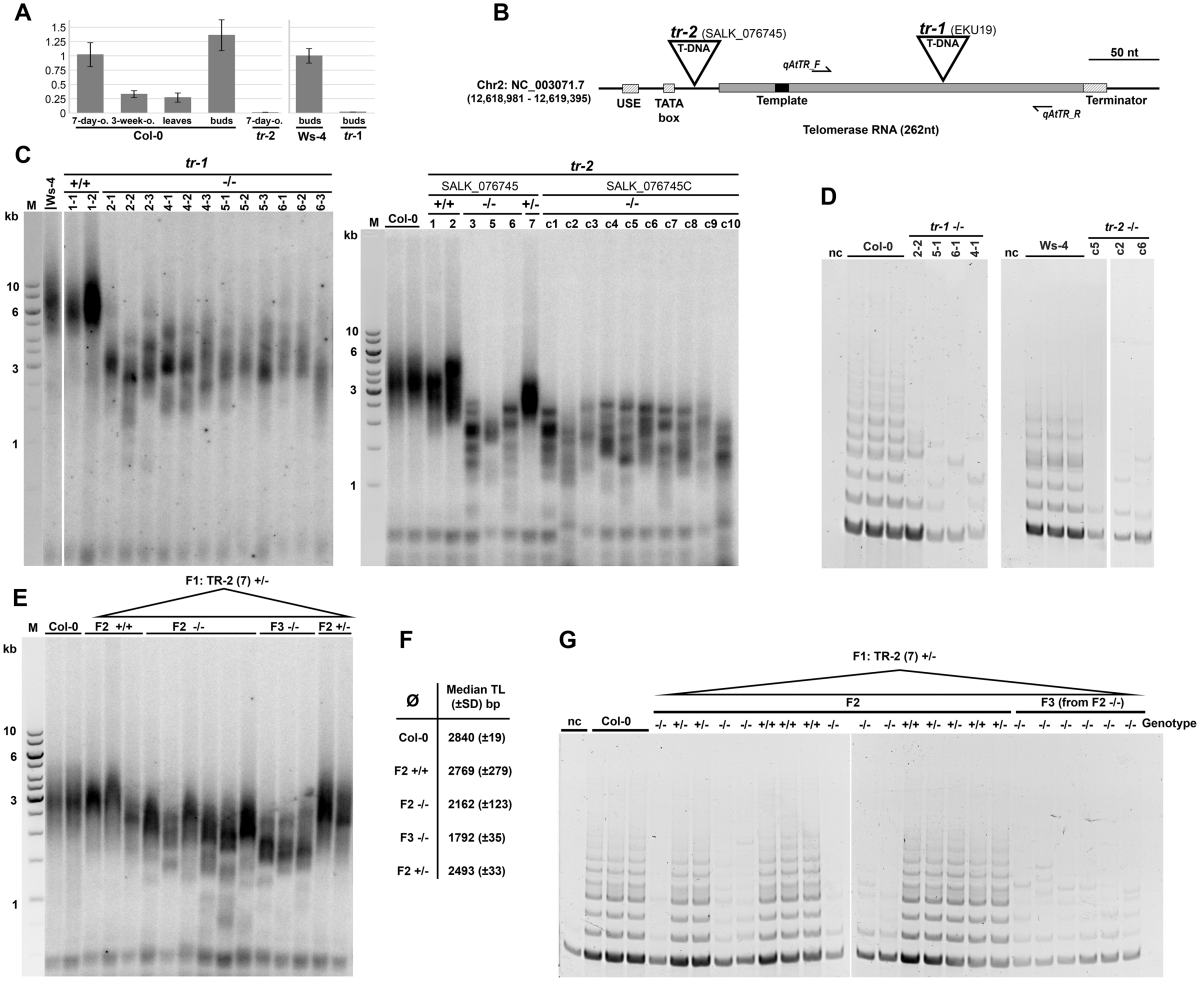
Analysis of *tr-1* (Ws background) and *tr-2* (Col) mutant plants shows loss of AtTR transcripts, telomere shortening, and telomerase dysfunction. (**A**) Relative AtTR transcript levels in tissues of *A. thaliana* (Col), and *tr-1* and *tr-2* lines. (**B**) Map of the *AtTR* region showing positions of T-DNA insertions in *tr-1* and *tr-2* mutants. (**C**) Analysis of terminal restriction fragments (TRF) in segregated wt (+/+) plants and homozygous (-/-) plants of the *tr-1* line (background Wassilevskija, Ws-4) (left panel). The right panel shows the corresponding results in the background wt *A. thaliana* (Col-0), segregated wt, and heterozygous and homozygous *tr-2* plants. Note the telomere shortening and telomere signal partitioning in both *tr-1* and *tr-2*. (**D**) TRAP assays of *tr-1* (left panel) and *tr-2* plants (right panel) show the loss of telomerase activity compared to wt plants of corresponding ecotypes. (**E**) TRF analysis of plants segregated from a heterozygous TR-2 (+/−) plant demonstrates a progressive telomere shortening in F2 and F3 homozygous mutants. (**F**) Median telomere lengths evaluated from results in panel E. (**G**) Telomerase activities in Col-0, and F2 and F3 plants segregated from *TR-2*(+/−). nc, negative control.

A natural tissue-specific expression of *AtTR* transcripts, especially in primary root and lateral root apices of 3-week-old seedlings and in cultured cells, was detected previously by whole-mount *in situ* hybridization (30). In the other tissue samples, no AtTR signal was found using northern hybridization (30). Using RT-qPCR, we found *AtTR* transcripts to be most abundant in 7-day-old seedlings and flower buds, and lower, yet detectable levels were observed in 3-week-old seedlings and young leaves (Figure 4A). Thus, the AtTR expression follows a tissue-specific pattern similar to that in *Allium* TR with a high expression in meristem tissues and low expression in leaves (see Figure 2B above). This pattern is typical also for expression of the telomerase protein subunit *AtTERT* (58). Moreover, we estimated the ratio of *AcTR*:*AcTERT* and *AtTR*:*AtTERT* transcript levels in seedlings as 64:1 and 75:1, respectively.

Another line of evidence supporting AtTR as the genuine TR subunit in *A. thaliana* comes from analyses of T-DNA insertion mutants in the *AtTR* gene. We found two available *attr* mutants—*tr*-*1* (Wassilevskija background) previously reported for the lack of *AtTR* transcripts in northern blot analysis (30), and *tr*-*2* (Columbia background) (see Figure 4B). The *tr*-*1* line harbours the T-DNA insertion in the transcribed region 146 bp downstream of the TSS, and in *tr*-*2* T-DNA is inserted within the promoter region of *AtTR*, 14 bp upstream of TSS. Our RT-qPCR analysis showed markedly decreased levels of AtTR transcript in both *tr-1* and *tr-2* mutant lines compared to the respective wild type seedlings (Figure 4A). Analyses of telomere lengths demonstrated a substantial telomere shortening in all homozygous mutant plants of both *attr* lines when compared to corresponding WT or heterozygous plants (Figure 4C). In addition to telomere shortening, partitioning of the natural TRF pattern, i.e. a single homogeneous smeared band of telomeric DNA scattered to multiple distinct signals, was observed in most *tr*-*1* mutant plants and in all *tr*-*2*, which is a TRF pattern typical for *attert* mutants (49,59,60) Moreover, both mutant lines revealed a complete loss or a considerable decrease of telomerase activity in TRAP assays (Figure 4D). To examine a possible trans-generational progression of telomere shortening, we propagated heterozygous *TR-2* (+/−) plant (F1 generation) and analysed telomere lengths in their progeny in subsequent F2 and F3 generations (Figure 4E). Indeed, gradual telomere shortening from parental *TR-2* (+/−) plant to homozygous F2 and F3 *TR-2* (−/−) plants results in a loss of ca. 400–600 bp per generation (Figure 4F) associated with the loss of telomerase activity, as demonstrated by TRF and TRAP analyses (Figure 4E, G, respectively), in contrast to the wild-type telomere length and telomerase activity observed in heterozygous (+/−) and segregated wild-type (+/+) F2 plants.

### Transgenic expression of AtTR restores telomerase activity and telomere maintenance in *attr* plants

**Figure 5.**
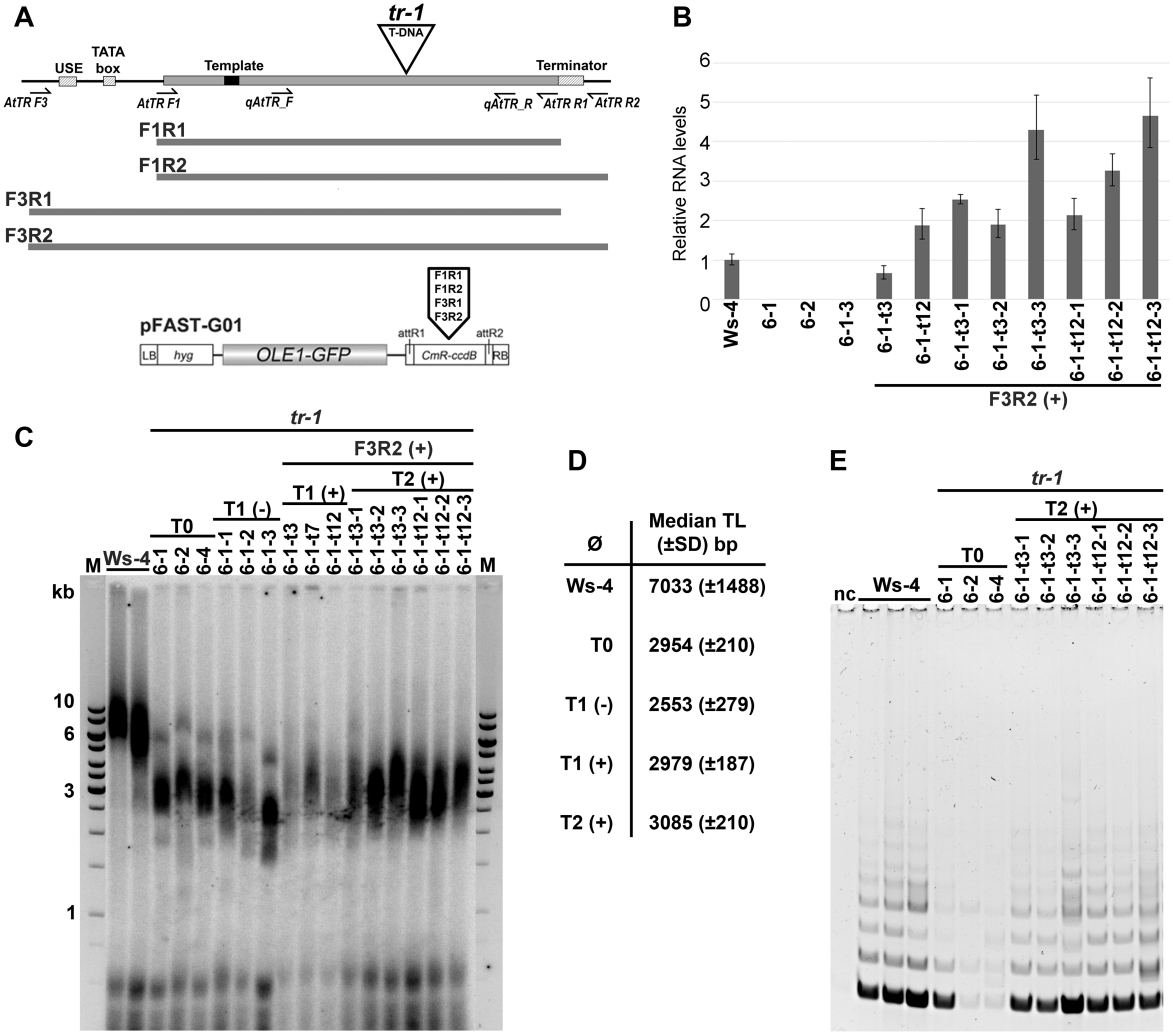
Complementation of telomerase function in *tr-1* plants. (**A**) Constructs used for *in vivo* complementation and primer positions. (**B**) relative transcript levels of TR in transformed *tr-1* plants compared to wt Ws-4 plants and parental *tr-1* plants. (**C**) TRF analysis in wt Ws-4 plants, parental T0 and progeny T1 *tr-1* plants, and corresponding T1 and T2 *tr-1* plants transformed with F3R2 constructs. Note the telomere signal partioning visible in T0 and T1(–) plants in contrast to complemented T1(+), T2(+) and wt Ws-4 plants. (**D**), Telomere stabilisation or slight elongation in complemented *tr-1* plants (T1(+), T2(+)) at the level of T0 plants can be seen in median lengths calculated from TRF data (panel C). Non-complemented T1(-) plants continue telomere shortening. (**E**) Telomerase activity is rescued in F3R2-complemented *tr-1* plants (T2(+)) to the level of wt plants (Ws-4), while T0 plants prior to transformation lack telomerase activity. nc, negative control.

To confirm the telomerase templating function of AtTR unequivocally, we transformed *tr -1* mutants with various *AtTR* constructs designed to cover the transcribed region of the *AtTR* gene only, or elongated with the terminator region, under the control of a native promoter or an artificial U6 promoter (Figure 5A). We analyzed telomerase activity and telomere length in the complemented plants with respect to the parental *tr-1* mutants used for transformation (T0). We found plants expressing the construct F3R2 were the most effective in reconstitution of telomerase activity (Supplementary Figure S5) and transformants displayed TR levels similar to natural *AtTR* transcripts (Figures 4B, 5B). Moreover, telomeres of T1 plants complemented with the F3R2 construct maintained their telomeres at a level similar or slightly longer than telomeres of parental G1 plants, while the corresponding G2 *tr-1* plants continued telomere shortening (Figure 5C,D). In the next generation of complemented plants (T2), telomere lengths remained stable, without any evident trans-generational change (Figure 5C,D) that is similar to results from *attert* mutants complemented with an *AtTERT* gene construct under control of a native promoter (61). Correspondingly, telomerase activity (Figure 5E) was recovered in the *AtTR*-complemented plants. We conclude that *AtTR* is able to complement defects in telomere stability and telomerase activity observed in *attr* plants *in vivo* and is the genuine telomerase RNA subunit in *A. thaliana*.

## DISCUSSION

### Monophyletic origin of telomerase RNA in land plants

Although the primary aim of this study was to identify TR subunits in plants of the *Allium* genus with a specific 12nt telomere repeat unit, we were also able to apply this knowledge to determine TR subunits in the other representative Asparagales species with either human-like or *Arabidopsis*-like telomere repeats. As the putative template regions in these TRs corresponded with the known telomere unit sequences, these results encouraged us to extend the TR identification across the land plant taxa, including numerous important crop plants. The outcome was very unexpected, as the existing paradigm in plant telomerase evolution is based on a relatively conserved TERT subunit, associating with TRs which are highly divergent in their sequences among even closely related species (57). However, our results demonstrate the ancient origin of plant TR genes which allowed us to identify these in representative taxa across land plants (Figure 1, Supplementary Figure S1). It has yet to be shown to which extent this homology is maintained in lower plant taxons to make solid conclusions on the relations of the evolution of plant telomerase RNAs to the early plant evolution and evolution of early eukaryotes in general. Interpretation is complicated by ongoing discussion about positions of the root of the eukaryotic phylogeny (12,62,63). Nevertheless, our results demonstrate that RNA Pol III-dependent synthesis of telomerase RNAs is not a specific and unique transition event specific within the ciliate lineage as previously suggested (27,64) and the general promoter architecture of plant TRs described here is similar to that of *Tetrahymena* TR and spliceosomal snRNAs (65). This is consistent with a hypothesis associating the origin of eukaryotes, linear chromosomes, telomeres and telomerase with invasion of self-splicing group II introns into archaeal hosts, giving rise to spliceosomal introns (66).

### Reconsideration of earlier views on plant TRs

The only plant telomerase RNA subunit candidate, *TER1*, has been previously reported in *Arabidopsis thaliana* (28). In that report, *TER1* could support telomerase function in overexpression and *in vitro* reconstitution experiments (28) but direct evidence of its *in vivo* function was missing. Together with *TER1*, its reshuffled paralog *TER2* and its variant splicing forms were also described, which did not show telomerase function. *TER1* partially overlaps the 5′-region of the *RAD52-1* gene, one of the two paralogs of RAD52 in Arabidopsis (67). The true role of *TER1* as telomerase subunit was questioned by the results of two studies soon after its description. First, telomere maintenance was not affected in the *rad52-1* mutant and *RAD52-1* RNAi knock-down plants, although *TER1* expression was reduced 6-fold and 3-fold, respectively (67). Second, a bioinformatic analysis of *TER1* loci among *Brassicaceae* demonstrated a striking sequence divergence within the template domain of putative *TER1* orthologs in plants which harbour canonical telomeres, thus not corresponding to the mutated *TER* template site. Orthologous *TER* genes that entirely lacked the template domain were found even in closely related species, including *Arabidopsis lyrata* (57). Moreover, we verified the sequence of the *TER1* gene with a divergent template motif (Supplementary Figure S6) that was speculated to exist in the Bela-1 ecotype of *A. thaliana* (68) although this ecotype possesses *Arabidopsis*-like telomeres as evidenced from genomic data (https://1001genomes.org/). Although these results did not directly exclude the possibility of *TER1* function in *A. thaliana*, they called for verification of *TER1* functionality as a telomerase RNA subunit. Indeed, our approach, entirely independent of the previous reports, allowed us to identify AtTR as the genuine telomerase RNA subunit and we demonstrate here with multiple lines of evidence that AtTR is the genuine telomerase RNA subunit in *A. thaliana*.

### Plant TRs are Pol III-dependent lncRNAs and associate with Dyskerin

Interestingly, AtTR has been characterized earlier as a hypoxic stress-responsive lncRNA transcribed by RNA polymerase III in *A. thaliana* (AtR8) and related *Brassicaceae* species (30,69). All its identified homologs possess the conserved USE, TATA, and Terminator elements. The authors also found that the transcription of AtR8 was Pol III-dependent and results in ca. 260 nt-long transcripts. Importantly, the transcript was missing in mutant plants deficient in the USE-binding transcription factor SNAPc (30). In addition to this description, we found that AtR8 and its orthologs from Brassicaceae (30,69) also harbour the previously unnoticed putative template region 5′-CTAAACCCT-3′ for synthesis of *Arabidopsis*-like telomeres. Our functional analyses of AtTR/AtR8, including *in vitro* reconstitution of telomerase activity with either a native or a mutant template region, analyses of telomeres and telomerase in *attr* mutants, and *in vivo* complementation of *attr* mutants with *AtTR* clearly demonstrated the templating function of AtTR in telomere synthesis. We thus conclude that AtTR is a Pol III-dependent lncRNA, and that its transcription is dependent on SNAPc transcription factor. As the structure of *TR* genes, including the promoter region, is conserved among the identified *TR* genes across land plants, this may hold true for the whole land plant phylogeny.

As we show in the case of *Allium* TR, the association of TRs with dyskerin appears to be conserved between plant and animal kingdoms which may facilitate future studies of plant telomerase complex assembly and purification for comparative structural studies. Such studies could possibly clarify a long-standing question - what specific steps, components and interactions involved in telomerase biogenesis are responsible for a reversible regulation of telomerase in plants (43), in contrast to its permanent postembryonic silencing in human somatic cells.

### *Allium* telomeres and telomerase RNA subunits—a difference that makes a difference

In his 1972 book, Steps to an Ecology of Mind, Gregory Bateson defined ‘information’ as a ‘difference that makes a difference’. In relation to this report, our studies on unusual *Allium* telomeres, markedly different from telomeres in the other higher eukaryotic taxa, yield useful information on several possible meanings of this definition. First, the unusually long *Allium* telomere repeat unit facilitated identification of candidate TRs in RNA-seq data of six *Allium* species as the first and key step of this study. The identified TRs showed mutual similarity at the sequence level, also outside of the template region, and subsequent functional examination of these TRs confirmed their templating role in telomere synthesis. Second, identification of the *Allium* TRs allowed us to elucidate the previously described evolutionary switches in telomere sequences of Asparagales at the molecular level. Importantly, the putative template region of the novel TRs in representative species of the Asparagales order with human- and *Arabidopsis*-type telomeres corresponded to telomere repeat sequences in the respective plants. Third, we were able to apply this knowledge also in the other land plants, including the dicotyledonous model plants *A. thaliana* and *N. sylvestris*, as well as in plants with unusual telomere repeats (*G. hispidula, C. elegans*). This last direction of our research resulted in the surprising discovery that the previously characterised putative telomerase RNA subunit of *Arabidopsis* (the only reported TR among plants so far) is not a functional TR *in vivo* and, instead, the AtTR identified based on its related *Allium* TR is the genuine templating telomerase subunit. Thus, our studies of ‘a different’ *Allium* telomerase brought novel ‘information’ and a substantially different view on plant telomerase RNAs and their evolution, showing in fact an unexpected similarity and common origin of TR subunits across a wide range of flowering plants. This not only changes the existing paradigm in the field of plant telomere biology, but also allows for potential biotechnology applications and enrichment of telomerase research with numerous new model systems which (i) differ in lifespan and developmental strategies; (ii) must efficiently cope with environmental changes and (iii) could be easily regenerated from their totipotent cells. The TR subunits described here, as well as a relatively easy way of identifying TRs from genomic or transcriptomic data for any other land plant species, brings this perspective significantly closer. In addition, ‘*It has not escaped our notice*’ that using our bioinformatic approach to identify TR orthologs, it is also possible to predict telomere DNA sequence in virtually any land plant species with unknown telomeres if transcriptomic or genomic data are available.

## DATA AVAILABILITY

RNA-seq data generated in this project are available at NCBI (BioProject PRJNA542932). Access to datasets used for identification of TRs described in this work is given in Supplementary Table S3.

## Supplementary Material

gkz695_Supplemental_Files
