# Supplementary material for: Telomerase RNAs in land plants
Source: Nucleic Acids Res. 2019 Aug 8;47(18):9842–56. doi: 10.1093/nar/gkz695 (PMC6765143; doi:10.1093/nar/gkz695)
Supplement: gkz695_Supplemental_Files [file gkz695_supplemental_files.zip › Supplementary Figures.pdf]

Supplementary Figures

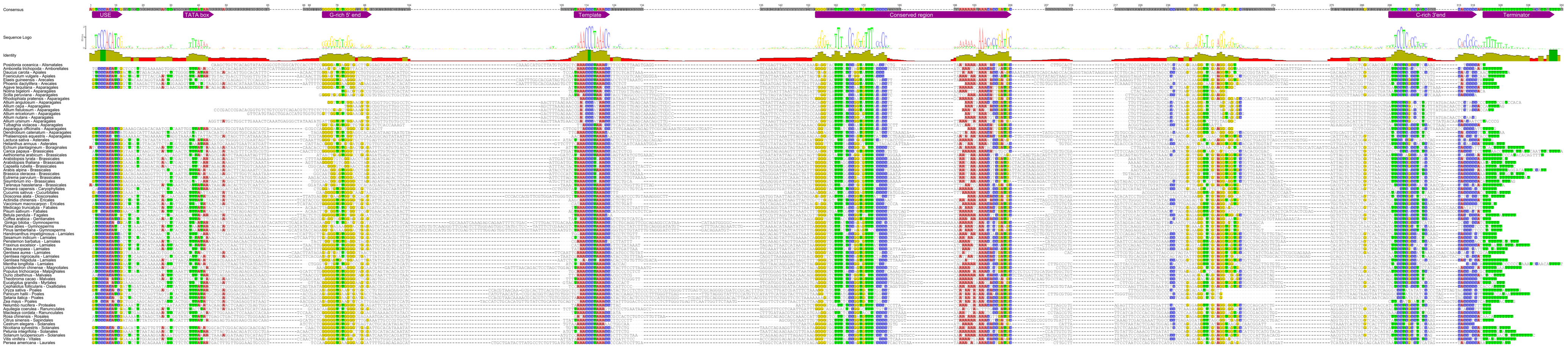

Supplementary Figure S1. Alignment of TRs identified in plants representing major clades across land plant phylogeny. In the upper part, the consensus sequence deduced from the alignment is depicted showing conserved regions of TR genes. Below the consensus, the level of nucleotide conservation along TRs is displayed as a sequence logo. The alignment of 75 identified TRs of diverse plants is shown below the sequence logo.

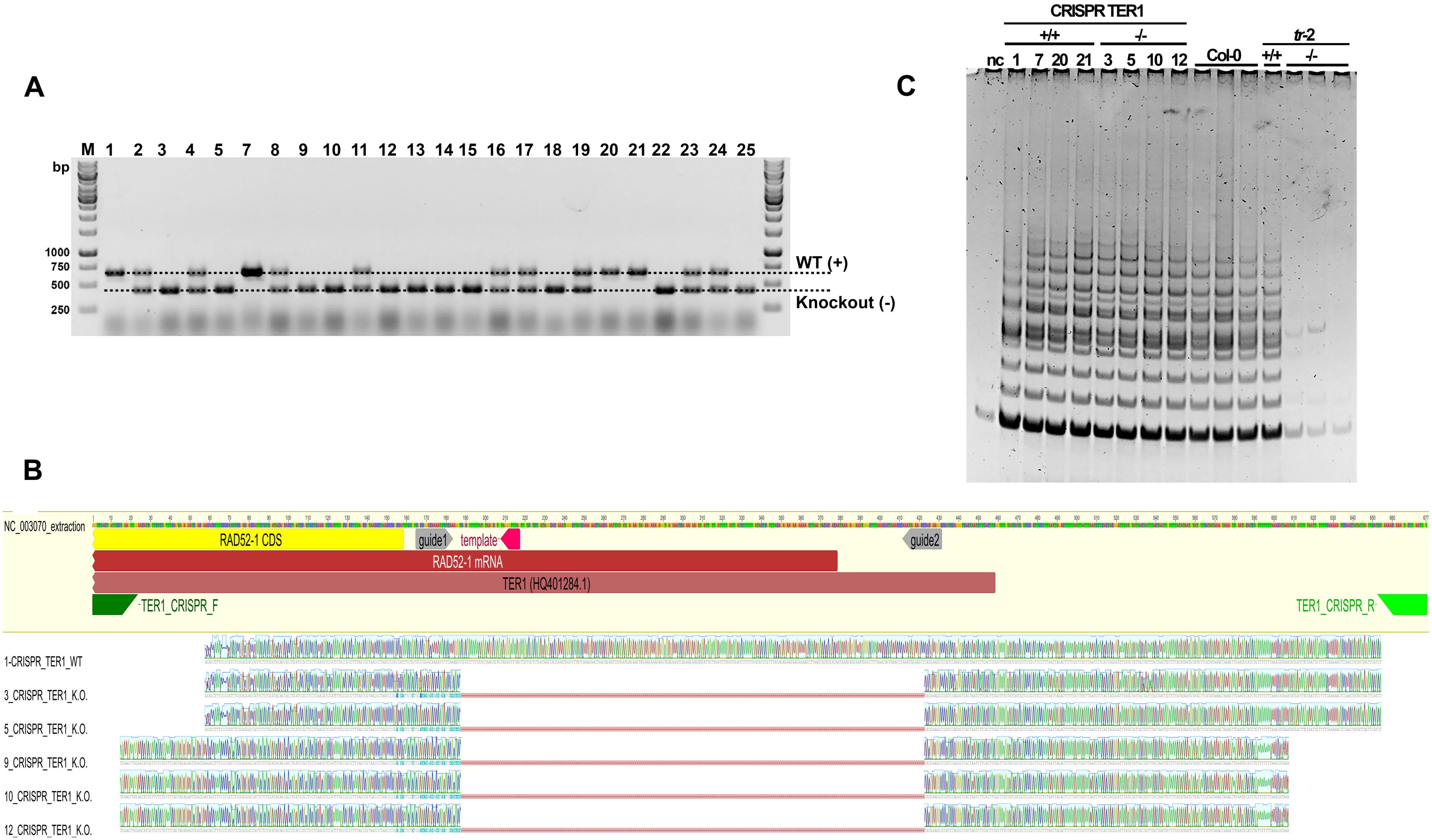

Supplementary Figure S2. CRISPR/Cas9 targeted TER1 deletion does not affect telomerase activity in *A. thaliana.* (**A**), genotyping of T3 of plants for homozygous deletion of *TER1.* (**B**), design of the targeted *TER1* deletion using the CRISPR/Cas9 system (upper part) and results of genotyping of homozygous wt and knockout plants using Sanger sequencing. (**C**), TRAP assays in TER1+/+ and TER1 -/-plants obtained from TER1 editing with CRISPR/Cas9, and their comparison to Col0 wt plants and segregating (+/+) and (-/-) *tr-2* plants. nc, negative control.

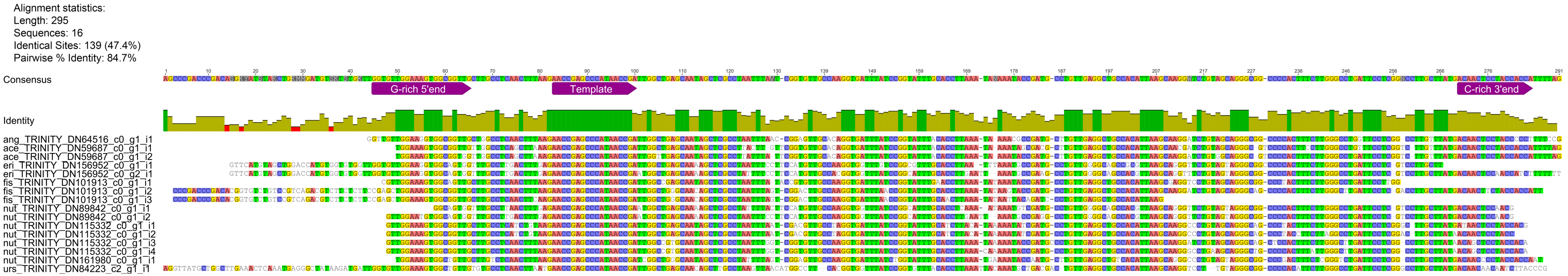

Supplementary Figure S3. Alignment of identified TRs in *Allium* species. Trinity transcripts are shown that shared sequence similarities in all datasets and thus presumably originated from *Allium* TR homologs

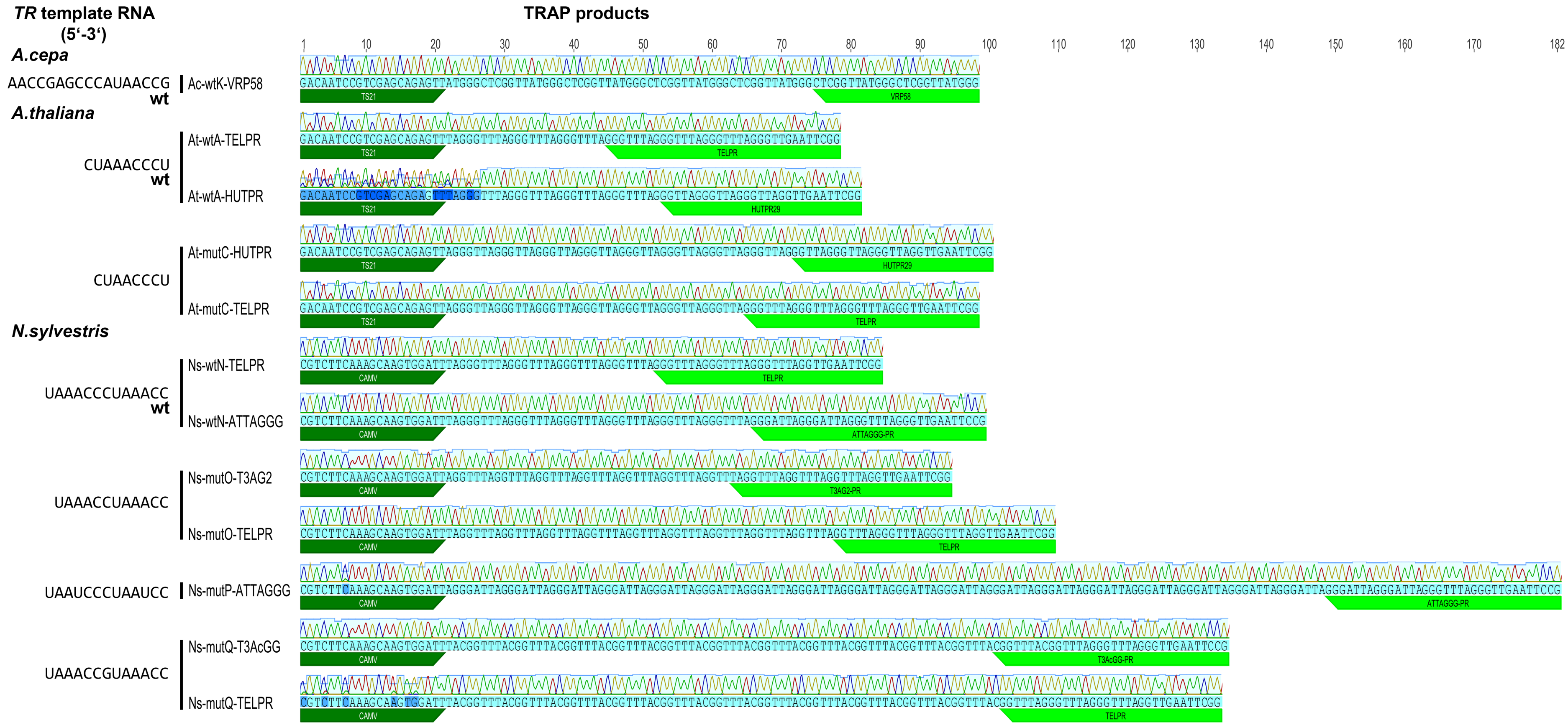

Supplementary Figure S4. Results of cloning and sequencing of TRAP products of telomerase *in vitro* reconstitutions with wt and mutant TRs with modified template regions. Sequences of template regions are displayed on the left, clone designations are next to these. Primers used in TRAP analyses are given at the borders of each of the cloned TRAP products.

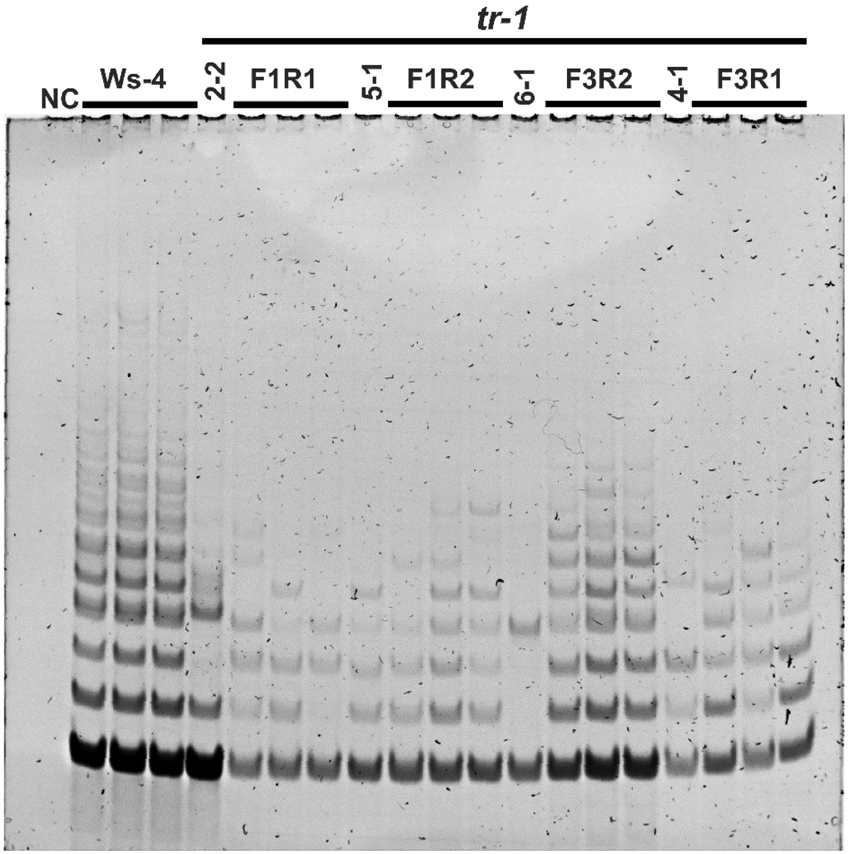

Supplementary Figure S5. TRAP assays in *tr-1* plants (2-2, 5-1, 6-1 and 4-1) before and after complementation with F1R1, F1R2, F3R2 and F3R1 constructs, as indicated above individual lanes. wt Ws-4 plants are used as positive controls, NC, negative control.

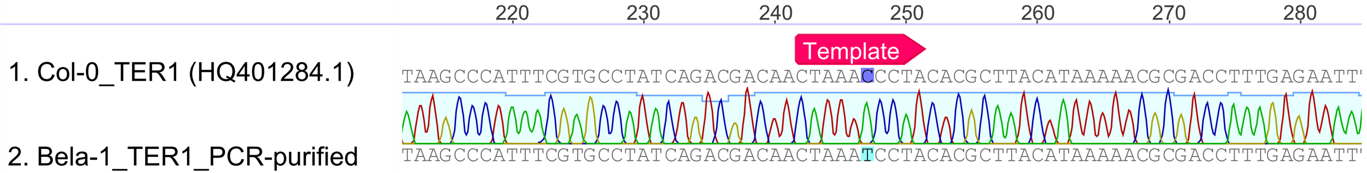

Supplementary Figure S6. The presumed template region of TER1 in *A. thaliana* (Bela-1 ecotype) cannot code for the Arabidopsis-type telomere. The result of sequencing of the PCR product of the *TER1* region in the Bela-1 ecotype (bottom line) is compared to the corresponding *TER1* region in Col-0 (the upper line).
