# Supplementary material for: Telomerase RNAs in land plants
Source: Nucleic Acids Res. 2019 Aug 8;47(18):9842–56. doi: 10.1093/nar/gkz695 (PMC6765143; doi:10.1093/nar/gkz695)
Supplement: gkz695_Supplemental_Files [file gkz695_supplemental_files.zip › Supplementary Tables.pdf]

Supplementary Table S1. Oligonucleotides used in the study.

**RNA subunit**		
AcTR		
AcTR_fw1	cloning, in vitro	TGGAAAGTGGCGGTGGTTG
AfTR_rev1	cloning, in vitro	AATGGTGGTAGAAGTTGTCATAA
SpTR		
SpTR_F	cloning, in vitro	CTTGGGGTGGGTGAAGTG
SpTR_R	cloning, in vitro	TCTTGGGGTGTTTGAGCAA
AtTR		
AtTR_FW1	cloning, in vitro	TTGGTCAAATAAGTTAAGGGGTGTGG
AtTR_REV1	cloning, in vitro	AAATATTTGGGGGTGGGAGGGTAA
AtTER1		
FAtTER1	cloning, in vitro	GAAGTGAAGATTAGTGACCCTGGAT
RAtTER1	cloning, in vitro	AATAATACACCAAAATGTCAAAACCAAAACC
AtCDT1a		
promCDT1a-Fw	cloning, in vitro	TCGTTCAGAGACAGCAATTCATAAT
promCDT1a-ATGRev	cloning, in vitro	GTGTACTCATTTTTATCAACAATGTCT
NsTR		
Nsylvestris-TR_Fw	cloning, in vitro	AACTCGGGGGTGTGGGGTTGAGATT
Nsylvestris-TR_Rev	cloning, in vitro	TTGGAGGTGCTTGGCAAGCGA
**RNA subunit mutagenesis**		
AtTRmut		
AtTRdelA	cloning	GTCTGCTTATTGATTGCTAACCCTGAACCCTCTCATGTTAAC
AtTRdelT	cloning	GTTAACATGAGAGGGTTCAGGGTTAGCAATCAATAAGCAGAC
NsTRmut		
NsTR mut delC	cloning	GCACATAAATATTGTTAAACCTAAACCACTTCTGTAACCAGAA
NsTR mut delG	cloning	TTCTGGTTACAGAAGTGGTTTAGGTTTAACAATATTTATGTGC
NsTR mut taatC3	cloning	GCACATAAATATTGTTAAtCCCTAATCCACTTCTGTAACCAGAAGG
NsTR mut attaG3	cloning	CCTTCTGGTTACAGAAGTGGATTAGGGATTAACAATATTTATGTGC
NsTR mut TA3CCg	cloning	GCACATAAATATTGTTAAACCGTAAACCACTTCTGTAACCAGAAGG
NsTR mut T3AcGG	cloning	CCTTCTGGTTACAGAAGTGGTTTACGGTTTAACAATATTTATGTGC
**TERT subunit**		
AcTERT		
Ac_ATG_fw-pEPEX	InFusion cloning	GGAGGCAGATACCATGGCAGGGAAAAGAAAACGC
Ac_Cend_rev-pEPEX	InFusion cloning	TAAGGGTAGCCCATGTTAAAATCTAATCTTCTGAAAGACAGA
NsTERT		
Nt_ATGFw1A_pEPEX	InFusion cloning	GGAGGCAGATACCATGTCGACGAAGAGACCGAGAGTT
Nt_TAG_Rev_pEPEX	InFusion cloning	TAAGGGTAGCCCATGGTTAATACTTAATCTTCCATAGTATAGAGGAACG
Nt_9exFw1CD	InFusion cloning	TTCCAAGCTTGTTAAGCTACGAAGG
Nt_9exRev3	InFusion cloning	TGCTTGAGCGTGAACTTCTCAAAC
SpTERT		
R_pEPEX_XbaI_pBSKS	InFusion cloning	ATCCACTAGTTCTAGAGGTATCTGCCTCCTTTGAGCG
F_pEPEX_XhoI_pBSKS	InFusion cloning	TACCGTCGACCTCGAGCAGTCTAGAGGATCTGGTTACCACTA
AtTERT		
F_pEPEX_ClaI_pBSKS	InFusion cloning	GATATCAAGCTTATCGATCAGTCTAGAGGATCTGGTTACCACTA
R_pEPEX_BamHI-At_pBSKS	InFusion cloning	GCGGCATTTTGGATCCGGTATCTGCCTCCTTTGAGCG

AtTR constructs in planta

AtTR-FW1_pB1	Gateway cloning	AAAAAGCAGGCTACTTGGTCAAATAAGTTAAGGGGTGTGG
AtTR-FW3_pB1	Gateway cloning	AAAAAGCAGGCTACCACCAGTGACGAACAAGTTACA
AtTR-REV1_pB2	Gateway cloning	AGAAAGCTGGGTCAAATATTTGGGGGTGGGAGGGTAA
AtTR-REV2_pB2	Gateway cloning	AGAAAGCTGGGTCGACTACATTCACGCTTTTGGATA
promU6.26-Fw_pB1	Gateway cloning	AAAAAGCAGGCTACAAGCTTTCGTTGAACAACGGA
promU6.26-Fw+pBSKS	InFusion cloning	CAGGAATTCGATATCAAGCTTTCGTTGAACAACGGA
promU6.26-Rev	InFusion cloning	CAATCACTACTTCGACTCTAGC
AtTR-REV2+pBSKS	InFusion cloning	GTCGACGGTATCGATAAGCTGACTACATTCACGCTTTTGGATA
AtTR_REV1+pBSKS	InFusion cloning	GTCGACGGTATCGATAAGCTAAATATTTGGGGGTGGGAGGGTAA
AtTR-FW2+U6.26	InFusion cloning	AGAGTCGAAGTAGTGATTGGTGACGTCACCAGTGAC
AtTR-FW1+U6.26	InFusion cloning	AGAGTCGAAGTAGTGATTGGTCAAATAAGTTAAGGGGTGTGG

TRAP

TS21	TRAP, substrate	GACAATCCGTCGAGCAGAGTT
CAMV	TRAP, substrate	CGTCTTCAAAGCAAGTGGATT
TELPR	TRAP, reverse	CCGAATTCAACCCTAAACCCTAAACCCTAAACCC
HUTPR29	TRAP, reverse	CCGAATTCAACCTAACCCTAACCCTAACC
VRP58	TRAP, reverse	CCCATAACCGAGCCCATAACCGAG
T3AG2-PR	TRAP, reverse	CCGAATTCAACCTAAACCTAAACCTAAACCTA
T3ACGG-PR	TRAP, reverse	CGGAATTCAACCCTAAACCCTAAACCGTAAACCG
ATTAG3-PR	TRAP, reverse	CGGAATTCAACCCTAAACCCTAATCCCTAATCCC

Genotyping

INRA_LB4	genotyping	CGTGTGCCAGGTGCCCACGGAATAGT
LBb1.3	genotyping	ATTTTGCCGATTTCGGAAC
genoAtTR_F	genotyping	CTCCGCCATTATTTTTCGTTAGCT
genoAtTR_R	genotyping	TTGAAGTCTCATCGGAAGGCTTAA

Crispr-TER1 mutants

TER1_CRISPR_F	genotyping	GGTCTACTGATTCCTGAAGTTGG
TER1_CRISPR_R	genotyping	ACAATTCAAGCACTTTCGTTTTCAG

RT-qPCR

qAcTR_F	qPCR	TATTTATTCGGTGTTGCACAGGTG
qAcTR_R	qPCR	CATAAACAAGACCGAGGAATCAGG
AcACT_F	qPCR	CAGGAGTTATGGTTGGAATGG
AcACT_R	qPCR	AGCACAGGATGTTCTTCA
qAcTERT_F	qPCR	ATATTGGCTAACGCAAAAGCACCT
qAcTERT_R	qPCR	TGTATCCGAATACAGAAGATCCTAATGC
qAtTR_F	qPCR	TTACTGGGGGTCTTAGGCCG
qAtTR_R	qPCR	GGAGGGTAAGGCGAGGAAAC
ubq_Fw	qPCR	AACGGGAAAGACGATTAC
ubq_Rev	qPCR	ACAAGATGAAGGGTGGAC

Supplementary Table S2: Statistics of Telomerase RNA candidates

Species	genome size (1C pg)	NGS raw reads	TRINITY	telomere repeat (length)	candidates with template region	shared sequences in all *Allium* datasets	TERT transcript
A. angulosum	15,1	151011864	366609	CTCGGTTATGGG (12)	24	1	+
A. cepa	16,75	64518048	210240	CTCGGTTATGGG (12)	5	2	+
A. ericetorum	?	155932784	529407	CTCGGTTATGGG (12)	22	2	+
A. fistulosum	15,53	127291690	386217	CTCGGTTATGGG (12)	25	3	+
A. nutans	22,63	105013146	380366	CTCGGTTATGGG (12)	28	7	+
A. ursinum	30,17	145956874	441843	CTCGGTTATGGG (12)	80	1	+
C. elegans	9,76	95463938	338591	TTTTTTAGGG (10)	2339	not defined	+
S. peruviana	?	66865026	476296	TTAGGG (6)	60903	not defined	+
T. violacea	19,83	101341874	430607	TTAGGG (6)	65997	not defined	+

Supplementary Table S3: TRs identified in datasets from representative monocot, eudicot and gymnospermsspecies

Order	Species	Accession (genomic/transcriptomic)	Assembly TR coordinates *or notes*	Strand	TR sequence 5'-3'
Alismatales	*Posidonia oceanica*	GFJT01022271.1	*GenBank TSA*	pos.	[illegible]
Amborellales	*Amborella trichopoda*	NW_006494910.1	7,781,382 - 7,781,726	pos.	[illegible]
Apiales	*Daucus carota*	NC_030389.1	27,698,097 - 27,698,445	neg.	[illegible]
Apiales	*Foeniculum vulgare*	PHNY01034214.1	1,639 - 1,976	pos.	[illegible]
Arecales	*Elaeis guineensis*	NC_025994.1	7,710,066 -7,710,410	pos.	[illegible]
Arecales	*Phoenix dactylifera*	NW_008249538.1	6,465 - 6,801	neg.	[illegible]
Asparagales	*Agave tequilana*	*SRR1503763 assembled reads	*GenBank SRA (SRR1503763)*	pos.	[illegible]
Asparagales	*Allium angulosum*	ang_TRINITY_DN64516_c0_g1_i1	https://www.ibp.cz/local/data/telomeraser	pos.	[illegible]
Asparagales	*Allium cepa*	ace_TRINITY_DN59687_c0_g1_i2	https://www.ibp.cz/local/data/telomeraser	pos.	[illegible]
Asparagales	*Allium ericetorum*	eri_TRINITY_DN156952_c0_g2_i1	https://www.ibp.cz/local/data/telomeraser	pos.	[illegible]
Asparagales	*Allium fistulosum*	fis_TRINITY_DN101913_c0_g1_i2	https://www.ibp.cz/local/data/telomeraser	pos.	[illegible]
Asparagales	*Allium nutans*	nut_TRINITY_DN115332_c0_g1_i1	https://www.ibp.cz/local/data/telomeraser	pos.	[illegible]
Asparagales	*Allium ursinum*	urs_TRINITY_DN84223_c2_g1_i1	https://www.ibp.cz/local/data/telomeraser	pos.	[illegible]
Asparagales	*Thulbagia violacea*	tul_TRINITY_DN12736_c0_g1_i1	https://www.ibp.cz/local/data/telomeraser	pos.	[illegible]
Asparagales	*Scilla peruviana*	sci_TRINITY_DN93444_c0_g2_i1	https://www.ibp.cz/local/data/telomeraser	pos.	[illegible]
Asparagales	*Nolina bigelovii*	RQZP_scaffold_2111914	*oneKP*	neg.	[illegible]
Asparagales	*Rhodophiala pratensis*	JDTY_scaffold_2072912	*oneKP*	neg.	[illegible]
Asparagales	*Asparagus officinalis*	NC_033796.1	55,677,063 - 55,677,362	neg.	[illegible]
Asparagales	*Dendrobium catenatum*	NW_018285661.1	32,785 - 33,119	pos.	[illegible]
Asparagales	*Phalaenopsis equestris*	NW_018251343.1	150,474 - 150,810	neg.	[illegible]
Asterales	*Helianthus annuus*	NC_035435.1	67,823,458 - 67,823,788	neg.	[illegible]
Asterales	*Lactuca sativa*	NW_019679676.1	1,407,647 - 1,407,986	neg.	[illegible]
Boraginales	*Echium plantagineum*	QFAX01000791.1	235,097 - 235,426	pos.	[illegible]
Brassicales	*Aethionema arabicum*	KE151693.1	19,420 - 19,768	pos.	[illegible]
Brassicales	*Arabidopsis lyrata*	NW_003302552.1	13,526,294 - 13,526,630	pos.	[illegible]
Brassicales	*Arabidopsis thaliana*	NC_003071.7	12,618,999 - 12,619,340	pos.	[illegible]
Brassicales	*Arabis alpina*	KL981859.1	6,871 - 7,221	pos.	[illegible]
Brassicales	*Brassica oleracea*	NC_027751.1	45,321,956 - 45,322,302	pos.	[illegible]
Brassicales	*Capsella rubella*	NW_006238920.1	7174949 - 7175293	pos.	[illegible]
Brassicales	*Eutrema parvulum*	CM001190.1	12,955,756 - 12,956,104	neg.	[illegible]
Brassicales	*Sisymbrium irio*	KE156162.1	139,412 - 139,754	neg.	[illegible]
Brassicales	*Carica papaya*	NW_019014633.1	1,242,494 - 1,242,837	neg.	[illegible]
Brassicales	*Tarenaya hassleriana*	NW_010971389.1	564,826 - 565,159	neg.	[illegible]
Caryophyllales	*Drosera capensis*	LIEC01011016.1	39,227 - 39,573	pos.	[illegible]
Cucurbitales	*Cucumis sativus*	NC_026657.1	4,387,804 - 4,388,095	pos.	[illegible]
Dioscoreales	*Dioscorea alata*	CZHE02043599.1	2,110 - 2,465	neg.	[illegible]
Ericales	*Vaccinium macrocarpon*	JOTO01168082.1	1,847 - 2,164	pos.	[illegible]
Ericales	*Actinidia chinensis*	AONS01000841.1	20,929 - 21,248	neg.	[illegible]
Fabales	*Medicago truncatula*	NC_016414.2	22,764,035 - 22,764,369	neg.	[illegible]
Fabales	*Pisum sativum*	PUCA012780952.1	2,378 - 2,721	pos.	[illegible]
Fagales	*Betula pendula*	FXXK01001271.1	19,177 - 19,501	pos.	[illegible]
Gentianales	*Coffea arabica*	NC_039914.1	3,676,658 - 3,676,995	neg.	[illegible]
Gymnosperms	*Ginkgo biloba*	*SRR8185327 assembled reads	*GenBank SRA (SRR8185327)*	pos.	[illegible]
Gymnosperms	*Picea abies*	CBVK0101923023.1	6,762 - 7,180	neg.	[illegible]
Gymnosperms	*Pinus lambertiana*	LMTP010003768.1	299,760 - 300,171	pos.	[illegible]
Lamiales	*Olea europaea*	UWJE01011027.1	272,907 - 273,237	neg.	[illegible]
Lamiales	*Penstemon barbatus*	QOIQ01016615.1	12,938 - 13,263	neg.	[illegible]
Lamiales	*Fraxinus excelsior*	FTPI01000883.1	76,314 - 76,642	neg.	[illegible]
Lamiales	*Genlisea aurea*	KE528297.1	2,557 - 2,901	pos.	[illegible]
Lamiales	*Genlisea hispidula*	*ERR299255 assembled reads	*GenBank SRA (ERR299255)*	pos.	[illegible]
Lamiales	*Genlisea nigrocaulis*	*ERR412881 assembled reads	*GenBank SRA (ERR412881)*	pos.	[illegible]
Lamiales	*Handroanthus impetiginosus*	NKXS01000004.1	1,983 - 2,313	pos.	[illegible]
Lamiales	*Mentha longifolia*	LSBG01159436.1	1,082 - 1,413	neg.	[illegible]
Lamiales	*Sesamum indicum*	NC_026159.1	2,316,586 - 2,316,914	neg.	[illegible]
Laurales	*Persea americana*	NXHZ01004810.1	45,720 - 46,077	pos.	[illegible]
Magnoliales	*Liriodendron chinense*	PVNU02000262.1	764,723 - 765,075	pos.	[illegible]
Malpighiales	*Populus trichocarpa*	NC_037292.1	8,935,549 - 8,935,889	neg.	[illegible]
Malvales	*Durio zibethinus*	NW_019167871	10,632,292 - 10,632,627	pos.	[illegible]
Malvales	*Theobroma cacao*	NC_030859.1	13,601,850 - 13,602,183	neg.	[illegible]
Myrtales	*Eucalyptus grandis*	NW_010092444.1	32,948,650 - 32,948,968	neg.	[illegible]
Oxallidales	*Cephalotus follicularis*	BDDD01000524.1	142,300 - 142,631	neg.	[illegible]
Poales	*Panicum hallii*	NC_038044.1	4,092,116 - 4,092,491	neg.	[illegible]
Poales	*Oryza sativa*	NC_029267.1	7,440,546 - 7,440,910	neg.	[illegible]
Poales	*Setaria italica*	NC_028452.1	5,810,993 - 5,811,353	pos.	[illegible]
Poales	*Zea mays*	NC_024461.2	32079924 - 32,080,242	neg.	[illegible]
Proteales	*Nelumbo nucifera*	NW_010729090.1	6,366,676 - 6,366,973	neg.	[illegible]
Ranunculales	*Aquilegia coerulea*	KZ305027.1	5,544,346 - 5,544,666	neg.	[illegible]
Ranunculales	*Macleaya cordata*	MVGT01001400.1	97,473 - 97,786	pos.	[illegible]
Rosales	*Rosa chinensis*	CM009587.1	60,849,207 - 60,849,538	pos.	[illegible]
Sapindales	*Citrus sinensis*	NC_023054.1	5,889,437 - 5,889,760	neg.	[illegible]
Solanales	*Solanum lycopersicum*	NC_015444.3	59,261,478 - 59,261,822	pos.	[illegible]
Solanales	*Cestrum elegans*	ces_TRINITY_DN38755_c0_g1_i1	https://www.ibp.cz/local/data/telomeraser	pos.	[illegible]
Solanales	*Nicotiana sylvestris*	NW_009498048.1	77,672 - 77,990	neg.	[illegible]
Solanales	*Petunia inflata*	Peinf101Scf00571	2,731,305 - 2,731,631 *(solgenomics.net)*	neg.	[illegible]
Vitales	*Vitis vinifera*	NC_012011.3	6,845,912 - 6,846,243	pos.	[illegible]

Supplementary Table S4: Comparative analysis of transcripts occurence within *Allium* datasets

Transcripts with minimal template region identified with grep*		Presence of sequence homologs identified in BLASTN					
		A. angulosum	*A.cepa*	*A. ericetorum*	*A. fistulosum*	*A. nutans*	*A. ursinum*
A. angulosum	ang_TRINITY_DN11315_c0_g1_i1		-	-	+	+	-
	ang_TRINITY_DN173283_c0_g1_i1		-	-	-	-	+
	ang_TRINITY_DN64516_c0_g1_i1		+	+	+	+	+
	ang_TRINITY_DN74449_c0_g1_i4		-	-	-	-	+
	ang_TRINITY_DN82208_c1_g2_i11		-	-	+	+	-
	ang_TRINITY_DN82208_c1_g2_i12		-	-	+	+	-
	ang_TRINITY_DN82208_c1_g2_i14		-	-	+	+	-
	ang_TRINITY_DN82208_c1_g2_i3		-	-	+	+	-
	ang_TRINITY_DN82208_c1_g2_i5		-	-	+	+	-
	ang_TRINITY_DN82208_c1_g2_i6		-	-	+	+	-
	ang_TRINITY_DN82208_c1_g2_i8		-	-	+	+	-
	ang_TRINITY_DN82208_c1_g2_i9		-	-	+	+	-
A. cepa	ace_TRINITY_DN59687_c0_g1_i1	+		+	+	+	+
	ace_TRINITY_DN59687_c0_g1_i2	+		+	+	+	+
A. ericetorum	eri_TRINITY_DN10895_c0_g1_i1	-	-		+	+	+
	eri_TRINITY_DN111547_c1_g1_i1	-	-		-	-	+
	eri_TRINITY_DN140298_c0_g1_i1	-	-		+	-	+
	eri_TRINITY_DN156952_c0_g1_i1	+	+		+	+	+
	eri_TRINITY_DN156952_c0_g2_i1	+	+		+	+	+
	eri_TRINITY_DN172700_c6_g2_i1	-	-		+	-	+
	eri_TRINITY_DN172700_c6_g2_i10	-	-		+	-	+
	eri_TRINITY_DN172700_c6_g2_i3	-	-		+	-	+
	eri_TRINITY_DN172700_c6_g2_i4	-	-		+	-	+
	eri_TRINITY_DN172700_c6_g2_i6	-	-		+	-	+
	eri_TRINITY_DN173689_c0_g1_i2	-	-		+	-	-
	eri_TRINITY_DN282036_c0_g1_i1	-	-		+	+	+
	eri_TRINITY_DN305153_c0_g1_i1	-	+		+	+	+
	eri_TRINITY_DN49887_c0_g1_i1	-	-		+	-	-
	eri_TRINITY_DN58523_c0_g1_i1	-	-		+	+	+
A.fistulosum	fis_TRINITY_DN101913_c0_g1_i1	+	+	+		+	+
	fis_TRINITY_DN101913_c0_g1_i2	+	+	+		+	+
	fis_TRINITY_DN101913_c0_g1_i3	+	+	+		+	+
	fis_TRINITY_DN107097_c1_g1_i2	-	-	-		+	-
	fis_TRINITY_DN107097_c1_g1_i3	-	-	-		+	-
	fis_TRINITY_DN113234_c1_g3_i1	+	-	-		+	-
	fis_TRINITY_DN113234_c1_g3_i5	+	-	-		+	-
	fis_TRINITY_DN118194_c0_g1_i2	-	-	+		-	-
	fis_TRINITY_DN118194_c0_g1_i3	-	-	+		-	-
	fis_TRINITY_DN119559_c0_g1_i1	-	-	-		+	-
	fis_TRINITY_DN137039_c0_g1_i1	-	-	-		-	-
	fis_TRINITY_DN14141_c0_g1_i1	-	-	+		+	+
	fis_TRINITY_DN79612_c0_g1_i1	-	-	-		+	-
	fis_TRINITY_DN84169_c1_g1_i1	-	-	+		+	-
	fis_TRINITY_DN87381_c0_g1_i2	-	-	-		+	-
	fis_TRINITY_DN88847_c0_g1_i1	-	-	-		+	-
	fis_TRINITY_DN93728_c0_g1_i1	-	-	+		+	+
	fis_TRINITY_DN93728_c0_g1_i2	-	-	+		+	+
	fis_TRINITY_DN99686_c0_g2_i1	-	-	-		+	-
A. nutans	nut_TRINITY_DN111558_c0_g1_i1	-	-	-	+		+
	nut_TRINITY_DN113457_c1_g1_i1	-	-	+	+		+
	nut_TRINITY_DN115332_c0_g1_i1	+	+	+	+		+
	nut_TRINITY_DN115332_c0_g1_i2	+	+	+	+		+
	nut_TRINITY_DN115332_c0_g1_i3	+	+	+	+		+
	nut_TRINITY_DN115332_c0_g1_i4	+	+	+	+		+
	nut_TRINITY_DN119101_c1_g1_i1	-	-	+	+		-
	nut_TRINITY_DN120549_c0_g1_i1	-	-	-	+		-
	nut_TRINITY_DN134515_c0_g3_i2	-	-	-	+		-
	nut_TRINITY_DN137488_c0_g1_i1	+	-	-	+		-
	nut_TRINITY_DN137488_c0_g1_i5	+	-	-	+		-
	nut_TRINITY_DN137488_c0_g1_i6	+	-	-	+		-
	nut_TRINITY_DN137488_c0_g1_i7	+	-	-	+		-
	nut_TRINITY_DN161980_c0_g1_i1	+	+	+	+		+
	nut_TRINITY_DN162370_c0_g1_i1	-	-	-	+		-
	nut_TRINITY_DN182499_c0_g1_i1	-	-	-	+		-
	nut_TRINITY_DN215222_c0_g1_i1	-	-	+	+		+
	nut_TRINITY_DN227439_c0_g1_i1	-	-	-	+		-
	nut_TRINITY_DN89842_c0_g1_i1	+	+	+	+		+
	nut_TRINITY_DN89842_c0_g1_i2	+	+	+	+		+
A.ursinum	urs_TRINITY_DN84223_c2_g1_i1	+	+	+	+	+	

* Only transcripts that show similarity with candidates from at least one *Allium* dataset are presented
